# Circulating extracellular vesicles in human cardiorenal syndrome promote renal injury in a kidney-on-chip system

**DOI:** 10.1172/jci.insight.165172

**Published:** 2023-11-22

**Authors:** Emeli Chatterjee, Rodosthenis S. Rodosthenous, Ville Kujala, Priyanka Gokulnath, Michail Spanos, Helge Immo Lehmann, Getulio Pereira de Oliveira, Mingjian Shi, Tyne W. Miller-Fleming, Guoping Li, Ionita Calin Ghiran, Katia Karalis, JoAnn Lindenfeld, Jonathan D. Mosley, Emily S. Lau, Jennifer E. Ho, Quanhu Sheng, Ravi Shah, Saumya Das

**Affiliations:** 1 Cardiovascular Research Center, Massachusetts General Hospital, Boston, Massachusetts, USA.; 2 Institute for Molecular Medicine Finland, Helsinki Institute of Life Science, University of Helsinki, Helsinki, Finland.; 3 Emulate, Inc., Boston, Massachusetts, USA.; 4 Department of Anesthesia, Beth Israel Deaconess Medical Center, Boston, Massachusetts, USA.; 5 Department of Biomedical Informatics and; 6 Department of Medicine, Vanderbilt University Medical Center, Nashville, Tennessee, USA.; 7 Regeneron Pharmaceuticals, Inc., Tarrytown, New York, USA.; 8 Cardiovascular Institute, Division of Cardiovascular Medicine, Department of Medicine, Beth Israel Deaconess Medical Center, Boston, Massachusetts, USA.; 9 Department of Biostatistics and; 10 Vanderbilt Translational and Clinical Research Center, Cardiology Division, Vanderbilt University Medical Center, Nashville, Tennessee, USA.

**Keywords:** Cardiology, Nephrology, Fibrosis, Heart failure, Noncoding RNAs

## Abstract

**BACKGROUND:**

Cardiorenal syndrome (CRS) — renal injury during heart failure (HF) — is linked to high morbidity. Whether circulating extracellular vesicles (EVs) and their RNA cargo directly impact its pathogenesis remains unclear.

**METHODS:**

We investigated the role of circulating EVs from patients with CRS on renal epithelial/endothelial cells using a microfluidic kidney-on-chip (KOC) model. The small RNA cargo of circulating EVs was regressed against serum creatinine to prioritize subsets of functionally relevant EV-miRNAs and their mRNA targets investigated using in silico pathway analysis, human genetics, and interrogation of expression in the KOC model and in renal tissue. The functional effects of EV-RNAs on kidney epithelial cells were experimentally validated.

**RESULTS:**

Renal epithelial and endothelial cells in the KOC model exhibited uptake of EVs from patients with HF. HF-CRS EVs led to higher expression of renal injury markers (IL18, LCN2, HAVCR1) relative to non-CRS EVs. A total of 15 EV-miRNAs were associated with creatinine, targeting 1,143 gene targets specifying pathways relevant to renal injury, including TGF-β and AMPK signaling. We observed directionally consistent changes in the expression of TGF-β pathway members (BMP6, FST, TIMP3) in the KOC model exposed to CRS EVs, which were validated in epithelial cells treated with corresponding inhibitors and mimics of miRNAs. A similar trend was observed in renal tissue with kidney injury. Mendelian randomization suggested a role for FST in renal function.

**CONCLUSION:**

Plasma EVs in patients with CRS elicit adverse transcriptional and phenotypic responses in a KOC model by regulating biologically relevant pathways, suggesting a role for EVs in CRS.

**TRIAL REGISTRATION:**

ClinicalTrials.gov NCT03345446.

**FUNDING:**

American Heart Association (AHA) (SFRN16SFRN31280008); National Heart, Lung, and Blood Institute (1R35HL150807-01); National Center for Advancing Translational Sciences (UH3 TR002878); and AHA (23CDA1045944)

## Graphical abstract

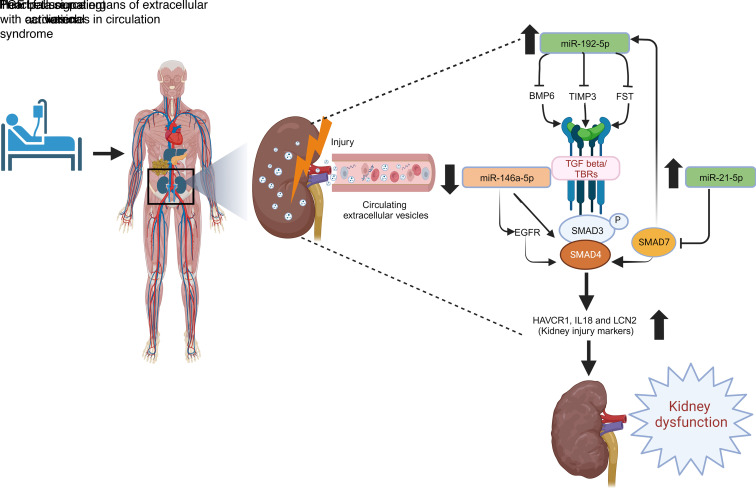

## Introduction

Kidney function is critical to cardiovascular homeostasis. The ability of the kidneys to respond to physiologic and pharmacologic inputs during states of fluid excess or decreased perfusion (e.g., heart failure, HF) is critical to symptomatic relief and prognosis (1). Although renal dysfunction in patients with HF (CRS) is associated with adverse outcomes (2, 3), therapies against renal hemodynamics (e.g., adenosine receptor agonists, ref. 4, or ultrafiltration for fluid unloading, refs. 5, 6) have limited benefits (5). Moreover, renal dysfunction is a risk factor for and develops during therapy in HF with preserved ejection fraction (HFpEF) and reduced ejection fraction (HFrEF), suggesting the importance of renal reserve across the hemodynamic spectrum. Indeed, despite a mechanistic and therapeutic focus on HFrEF, CRS in HFpEF is equally prevalent, is adverse (7, 8), and remains poorly understood. Recent advances in other disease conditions (e.g., cancer, ref. 9, and diabetes, ref. 10) suggest that trans-organ signaling via circulating extracellular vesicles (EVs) carrying molecular cargo (RNAs, proteins) may be an important mechanism of pathogenesis of different metabolic diseases. Advanced renal dysfunction is potentially associated with an increase in EVs bearing pro-inflammatory cargo (11); however, whether these EVs are causal in worsening renal function is clinically relevant but unknown. If circulating EVs in HF promote renal injury, studying their contents not only will unravel potential pathways of renal dysfunction in HF but may also open new therapeutic avenues directed at EV-based cargo to maintain renal function during HF therapy and improve outcomes. Nevertheless, the ability to study the functional effects of EVs in vivo remains limited, given the inability to directly assess the effect of EVs on the human kidney in a dynamic fashion and limited translatability of murine models of human renal disease (12, 13). While kidney organoids obtained from human induced pluripotent stem cells (hiPSCs) model human genetic renal disease (14), the development/induction of hiPSCs into mature kidney-like organoids with mature structures and vascularity remains challenging.

**Figure 1:**
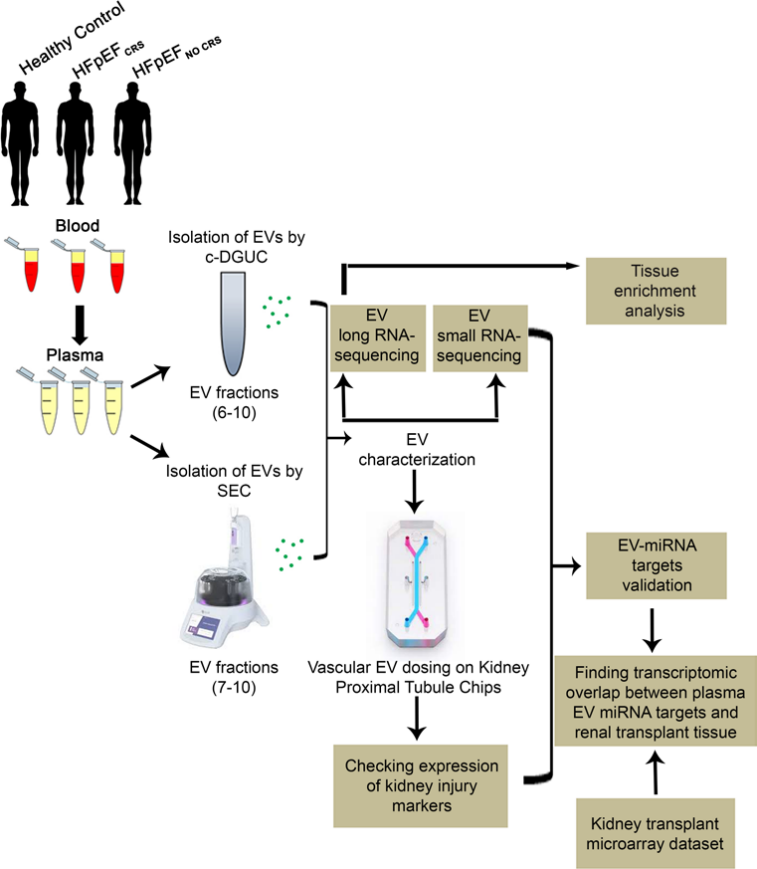
Study schema. c-DGUC, cushion gradient differential ultracentrifugation; SEC, size-exclusion chromatography.

Here, we utilize a recently described in vitro system (Emulate kidney-on-chip; ref. 15) that recapitulates key features of the blood-kidney interface (renal endothelial/epithelial cells) to test the hypothesis that EVs from individuals with HF with and without abnormal renal function induce molecular phenotypes of renal injury. We characterized plasma circulating EVs from patients with HFpEF with and without renal dysfunction (see Figure 1 for the overall study design). Using microfluidic perfusion to expose renal proximal tubular epithelial/endothelial cells on the kidney-on-chip (KOC) to EVs from patients with and without CRS, we examined the molecular phenotypic response in 2 ways: (i) expression of canonical markers of renal injury interleukin 18 (*IL18*) (16), lipocalin (*LCN2*) (17), and hepatitis A virus cellular receptor 1 (*HAVCR1*) (18) and function (*CST3*, cystatin C; ref. 19) and (ii) expression of computationally identified mRNA targets of HFpEF enriched EV-microRNAs from 2 sources — the renal cells on the chip (treated with EVs from HFpEF or controls) and human renal transplant biopsy samples with and without renal injury. Finally, the functional role of the EV-miRNAs was experimentally validated with gain- and loss-of-function approaches. Our primary findings implicate human HF-derived circulating EV cargo in transcriptional programs are central to renal injury (e.g., related to TGF-β and downstream pathways important for renal fibrosis) and support the translational impact of this emerging technology as a method to dissect renal responses.

## Results

### Characteristics of study samples.

Our study included 12 patients with HFpEF and 6 patients without HF (Healthy Control) to serve as controls. Of patients with HFpEF, 6 met criteria for CRS (HFpEF_CRS_). Baseline demographic and clinical characteristics are shown in Table 1 (HFpEF: 83% men with a high occurrence of coronary artery disease, hypertension, and diabetes). Patients with HFpEF_CRS_ were older (*P* < 0.01) and had increased levels of natriuretic peptides (markers of increased hemodynamic stress) on admission (*P* = 0.01) relative to patients with HFpEF_NO CRS_. In our small RNA-sequencing (RNA-Seq) cohort (9 HFpEF; 9 Healthy Control), patients with HFpEF were older and had poorer renal function (Table 2). Our long RNA-Seq cohort included 18 patients with HFpEF, 9 with high creatinine (1.1–1.6 mg/dL) and 9 with low creatinine (0.7–0.9 mg/dL) (Supplemental Table 1; supplemental material available online with this article; https://doi.org/10.1172/jci.insight.165172DS1).

### Isolation and characterization of circulating EVs.

**Figure 2:**
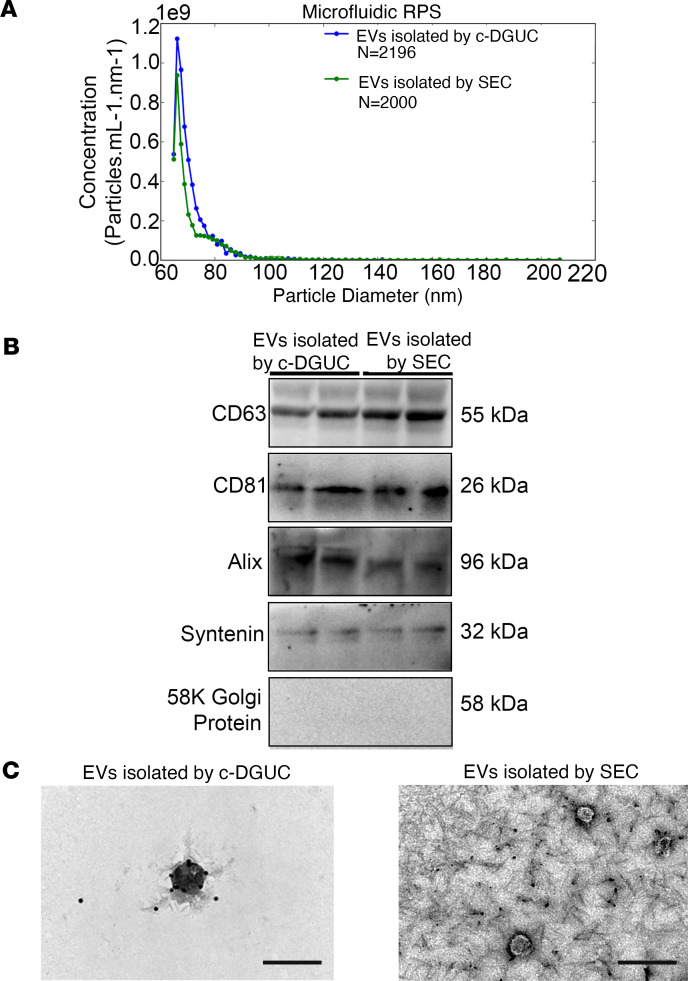
Characterization of EVs from human plasma. (**A**) Representative microfluidic resistive pulse sensing showing concentration and size distribution profiles of the EV population isolated by c-DGUC and SEC. (**B**) Representative Western blot of the expression of CD63, CD81, Alix, Syntenin, and 58K Golgi protein, as determined in the pooled EV samples isolated by both c-DGUC and SEC. (**C**) EVs isolated by both c-DGUC and SEC were visualized using TEM (scale bar used = 200 nm). Full-length, uncut gels are published in the online supplemental material.

To enhance the rigor of our study, we used 2 methods (c-DGUC and SEC) for EV isolation from Healthy Control (*n* = 6 for c-DGUC, *n* = 3 for SEC), HFpEF_CRS_ (*n* = 6 for c-DGUC, *n* = 4 for SEC), and HFpEF_NO CRS_ (*n* = 6 for c-DGUC, *n* = 3 for SEC) participants. Plasma EV concentrations were similar between control and HFpEF patients (1.98 × 10^11^ EV particles/mL on average). Isolated EVs were subjected to quality control as specified by minimal information for studies of extracellular vesicles guidelines (20). EV particle number and size distribution were consistent with published morphometric parameters for EVs (particle sizes ≈ 65–100 nm in diameter for both c-DGUC and SEC, Figure 2A). Canonical EV surface markers (CD63, CD81) and cargo proteins (Alix, Syntenin) were present in pooled EV fractions from both methods, whereas 58K Golgi protein (an indicator of intracellular component contamination) was not found in EVs isolated by either c-DGUC or SEC (Figure 2B). Finally, transmission electron microscopy (TEM) validated EVs with typical cup-shaped morphology delimited by a double-layered membrane (Figure 2C).

### Successful EV dosing to an in silico KOC model results in differential expression of kidney injury markers.

**Figure 3:**
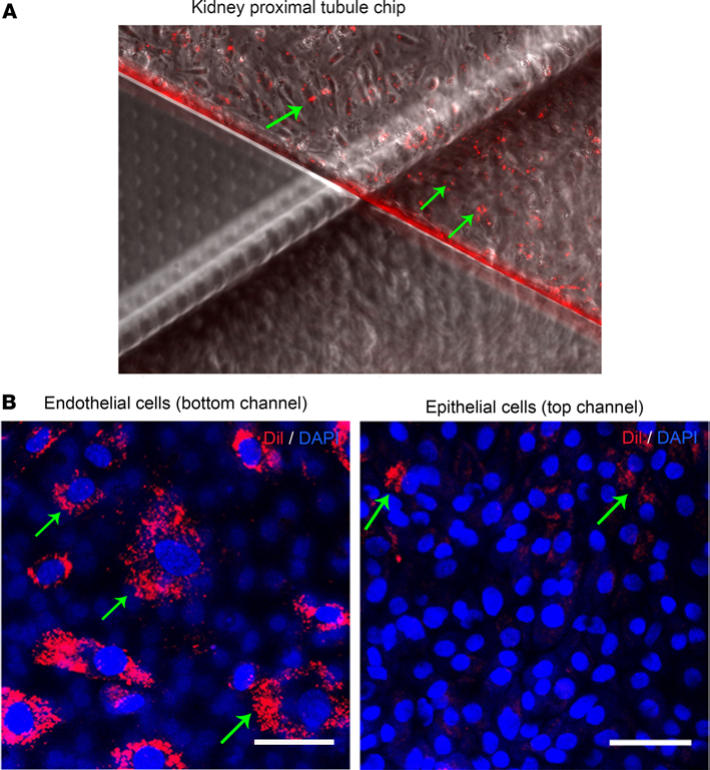
Successful dosing of EVs on KOC. Dil-stained EVs from a healthy control were visualized after 3-day perfusion period using fluorescence microscopy. (**A**) Representative images of the fluorescently labeled EVs (red), overlaid with a phase contrast image of the chip, mainly seen in the vascular endothelial (bottom) channel (scale bar = 100 μm). (**B**) Representative fluorescent confocal images of the EVs, cells in the vascular endothelial channel (bottom) and cells in the epithelial (top) channel (scale bar = 100 μm).

We next studied the effect of isolated EVs on the human KOC model. The KOC utilizes primary human cells to create a physiological model of the human proximal tubule with appropriate interface between epithelial and endothelial cells. EVs (final concentration 1.8 × 10^10^/mL after isolation) were perfused (total volume of perfusate 3 mL) for 72 hours on the KOC at a calculated exposure of 6,000 EVs/cell. Dil-labeled EVs from Healthy Control participants were observed after 3 days of EV perfusion through the vascular channel via fluorescence microscopy and were abundant within the endothelial (bottom) channel as well as within the epithelial (top) channel (Figure 3A), suggesting uptake of EVs by kidney cells across the chip. Confocal microscopy verified the abundant uptake of fluorescent EVs in the endothelial cells with lower uptake in the epithelial cells (Figure 3B). These results suggested effective delivery of EVs via microperfusion of the chip.

**Figure 4:**
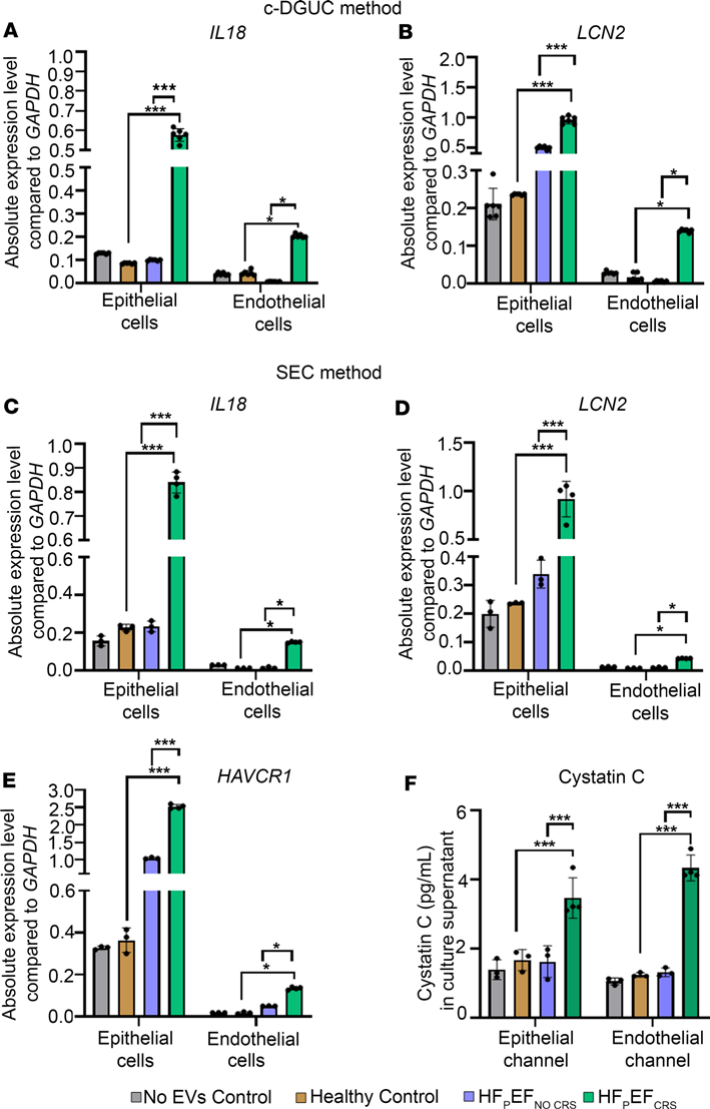
Differential expression of kidney injury marker genes and proteins in KOC model following 72 hours of incubation with EVs. (**A** and **B**) Increased mRNA expression of *IL18* (**A**) or *LCN2* (**B**) in the KOC cells treated with EVs from HFpEF_CRS_ compared with groups treated with EVs from HFpEF_NO CRS_ or Healthy Controls. “No EVs Control” KOC was exposed to PBS alone. EVs used for the treatment were isolated by c-DGUC. Three technical replicate chips were prepared for each biological replicate (*n* = 6) of each experimental group. (**C** and **D**) mRNA expression of *IL18* and *LCN2* were significantly increased in renal epithelial and endothelial cells of the KOC treated with HFpEF_CRS_ EVs compared with KOCs treated with EVs from HFpEF_NO CRS_ or Healthy Controls. (**E**) Increased mRNA expression of *HAVCR1* in the kidney cells treated with EVs from HFpEF_CRS_ compared with groups treated with EVs from HFpEF_NO CRS_ or Healthy Control. (**F**) Cystatin C ELISA showing higher expression in the group treated with EVs from HFpEF_CRS_ compared with groups treated with EVs from HFpEF_NO CRS_ or Healthy Control. EVs used for treatment (**C**–**F**) were isolated by SEC method. GAPDH was used as internal loading control for all quantitative RT-PCR experiments. Each biological replicate (*n* = 3 for Healthy Control and HFpEF_NO CRS_; *n* = 4 for HFpEF_CRS_) of each experimental group had 3 technical replicates (averaged for each data point). Results were analyzed by 1-way ANOVA with Tukey’s post hoc test and expressed as ±SEM of 3 independent experiments. *, *P* < 0.05; ***, *P* < 0.001.

Next, we sought to determine if EVs from HFpEF patients with CRS functionally affected the cells (at a concentration of 1.8 × 10^10^/mL) and then checked the effects of EVs specifically on epithelial cells. We assayed mRNA expression of 3 canonical markers of renal tubular injury relevant to clinical renal dysfunction: *IL18* (16), *LCN2* (17), and *HAVCR1* (18). The absolute expression of *IL18* mRNA was significantly higher in KOC-derived epithelial renal tubular cells treated with EVs from HFpEF_CRS_ relative to groups treated with EVs from HFpEF_NO CRS_ or Healthy Control participants and was consistent across EV isolation methodologies (Figure 4, A and C). *LCN2* and *HAVCR1* exhibited similar expression changes with HFpEF_CRS_ EVs (Figure 4, B, D, and E), with results consistent across the mode of EV isolation (c-DGUC and SEC; Figure 4, A–E). The quantitative PCR results were further corroborated by immunofluorescence studies that showed pronounced expression of HAVCR1, LCN2, and IL18 in the epithelial cells treated with the EVs from HFpEF_CRS_ group, compared with the Healthy Control group (Supplemental Figure 1). We assayed the protein expression of cystatin C, another biomarker of chronic kidney disease (constitutively expressed across cell types) in effluents coming from the KOC model (Figure 4F). Changes in cystatin C in the effluent may mimic changes in circulating cystatin C in vivo (and reflect changes in renal function) (19). Treatment with EVs from HFpEF_CRS_ significantly upregulated the effluent cystatin C expression of both endothelial and epithelial cells relative to other groups (Figure 4F). These results support an effect of HFpEF_CRS_ EVs on the transcriptional and functional state of the proximal nephron.

### EV small RNA cargo of HF patient–derived plasma EVs.

**Figure 5:**
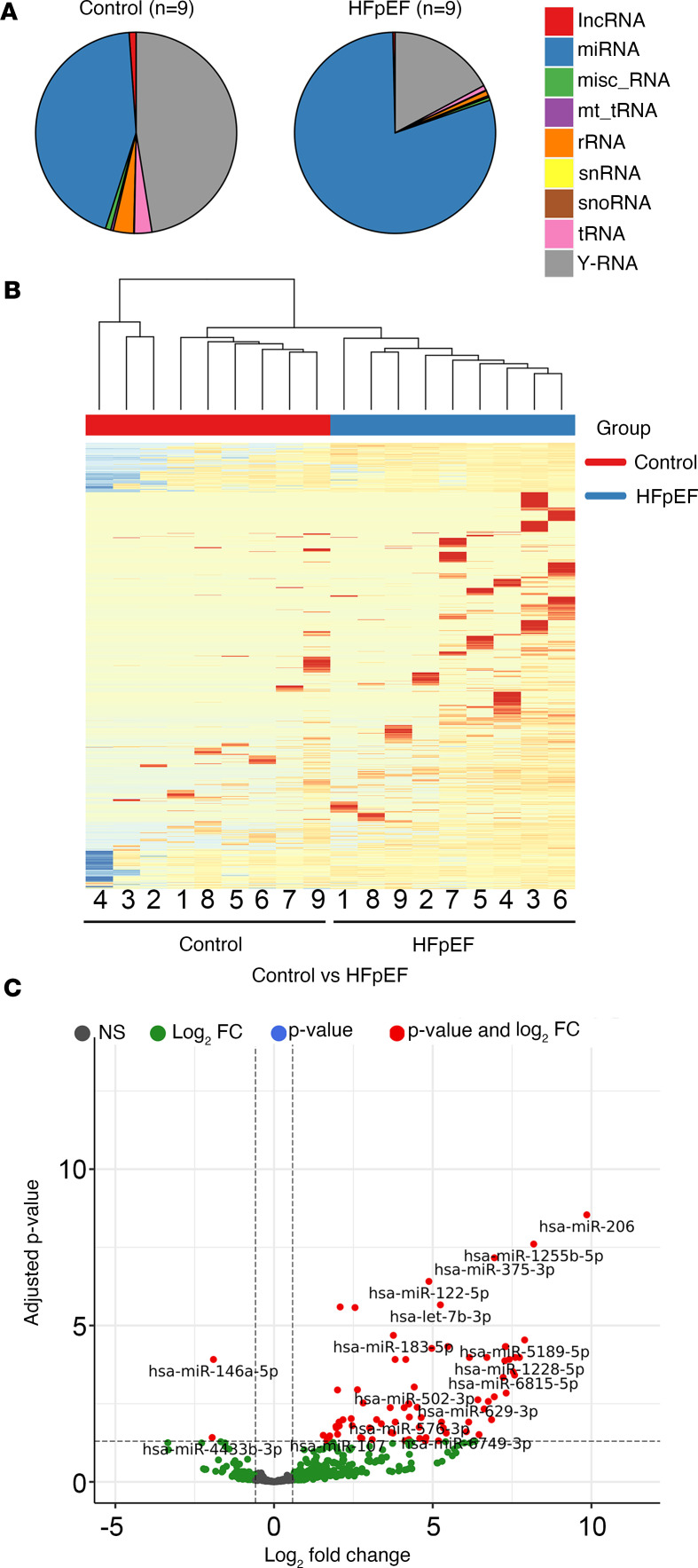
Summary of small RNA-Seq results. (**A**) Pie charts showing the differential distribution of noncoding RNAs according to RNA-Seq in 9 pairs of Healthy Control and HFpEF groups. lncRNA, long noncoding RNA; misc_RNA, miscellaneous RNA, mt_tRNA, mitochondrial tRNA; snRNA, small nuclear RNA; snoRNA, small nucleolar RNA. (**B**) Hierarchical clustering was performed for Healthy Control and HFpEF comparison (*n* = 9 for each group) based on the differentially expressed genes. The horizontal axis is composed of all the samples analyzed in the study, and vertical axis includes all differentially expressed genes. Top, control samples are denoted in red squares and HFpEF samples in blue squares. Dark blue to dark red color gradient illustrates lower to higher expression. (**C**) Volcano plot was created by all differentially expressed miRNAs. The *y* axis shows the adjusted *P* value, and the *x* axis displays the log_2_ fold-change value. The red dots represent the differentially expressed miRNAs with FDR-adjusted *P* ≤ 0.05 and absolute fold-change ≥ 1.5, while green dots represent nonsignificantly modulated miRNAs.

MiRNA cargo of EVs can be transferred across many cell types to affect the expression of target genes in recipient cells, as observed in many disease models (21, 22). We studied the extracellular small RNA transcriptome from 9 patients with HFpEF (with and without CRS) and 9 Healthy Controls, demonstrating marked differences in small RNA cargo (more miRNA reads, fewer Y-RNA [class of small noncoding RNA] reads; Figure 5A). After mapping reads to the genome (GENCODE GRCh38.p13), we detected 1,207 miRNAs. Overall, we observed systematic differences in the miRNA transcriptome of HFpEF EVs relative to that of Healthy Control participants (Figure 5B), with 78 differentially expressed miRNAs detected (at an absolute fold-change ≥ 1.5 and 5% FDR) between HFpEF and Healthy Control groups (Figure 5C).

### Source organs of circulating EVs revealed by deconvolution analysis.

**Figure 6:**
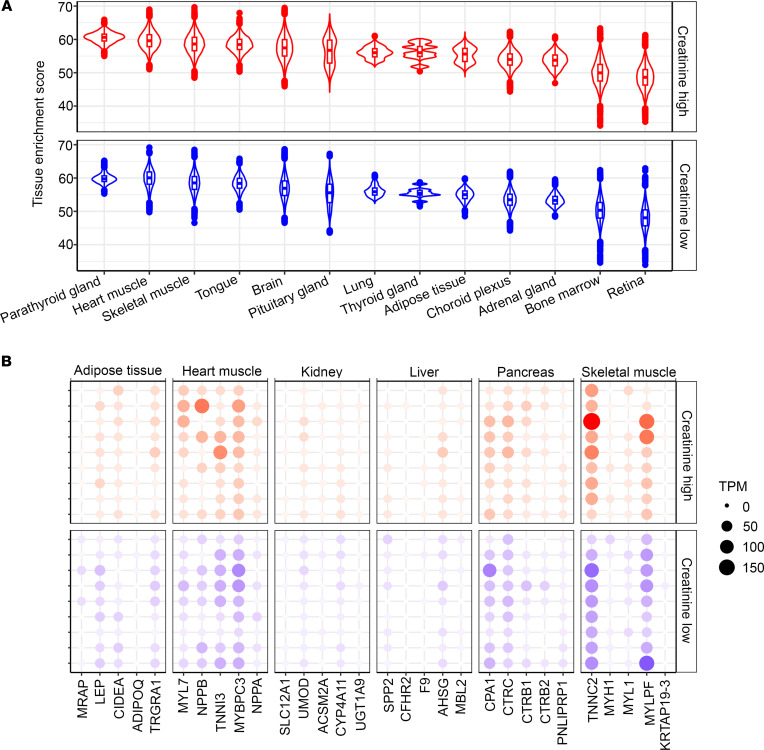
Tissue enrichment analysis using long RNA EV transcriptome. (**A**) Violin plot showing the tissue enrichment of the topmost upregulated transcripts in creatinine-high (red) and creatinine-low (blue) plasma EVs. (**B**) Dot plot expression of the top 6 enriched tissues with their respective tissue-specific transcripts in creatinine-high (red) and creatinine-low (blue) plasma EVs. TPM, transcripts per million.

We next wanted to investigate the source organs of these circulating EVs by analyzing the possible tissue origin of the EV-RNA transcripts. As miRNA expression across tissue types is promiscuous, we performed long RNA-Seq of plasma EVs from 9 creatinine-high (a clinical marker of renal dysfunction) and 9 creatinine-low patients with HFpEF and deconvoluted the results using the Human Protein Atlas (https://www.proteinatlas.org) to identify the tissue sources of the circulating EVs. The tissues were ranked based on the median of tissue enrichment scores of samples in the creatinine-high and -low groups, and these rankings were visualized using a violin plot in Figure 6A. There was a wide distribution of tissue sources for plasma EVs in HFpEF with representation of EVs from heart muscle, kidney, liver, pancreas, adipose tissue, and skeletal muscle (visualized using a dot plot for the top 5 genes with the highest tissue specificity scores, Figure 6B). There was no significant difference in the EV source between the 2 groups.

### Targeted pathway analyses converge on dysregulation of the TGF-β pathway in CRS.

**Figure 7:**
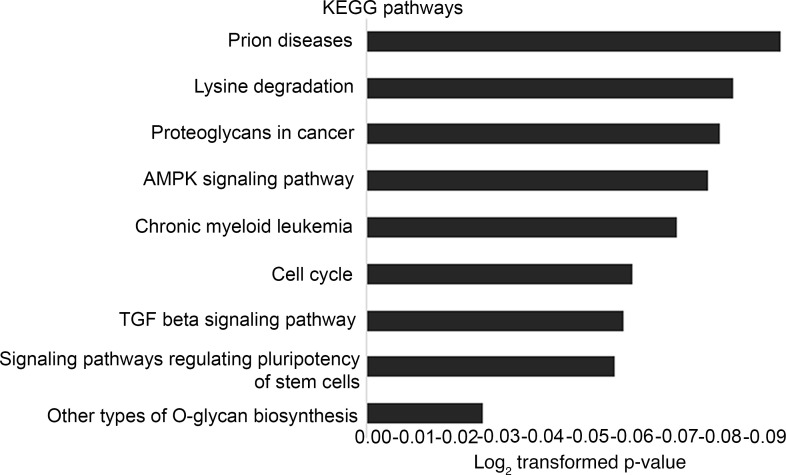
Comparative pathway analysis. Bar chart representing 9 most prominent pathways enriched in quantiles with differential EV-miRNA patterns in HF compared with Healthy Controls, as revealed by KEGG biological processes.

**Figure 8:**
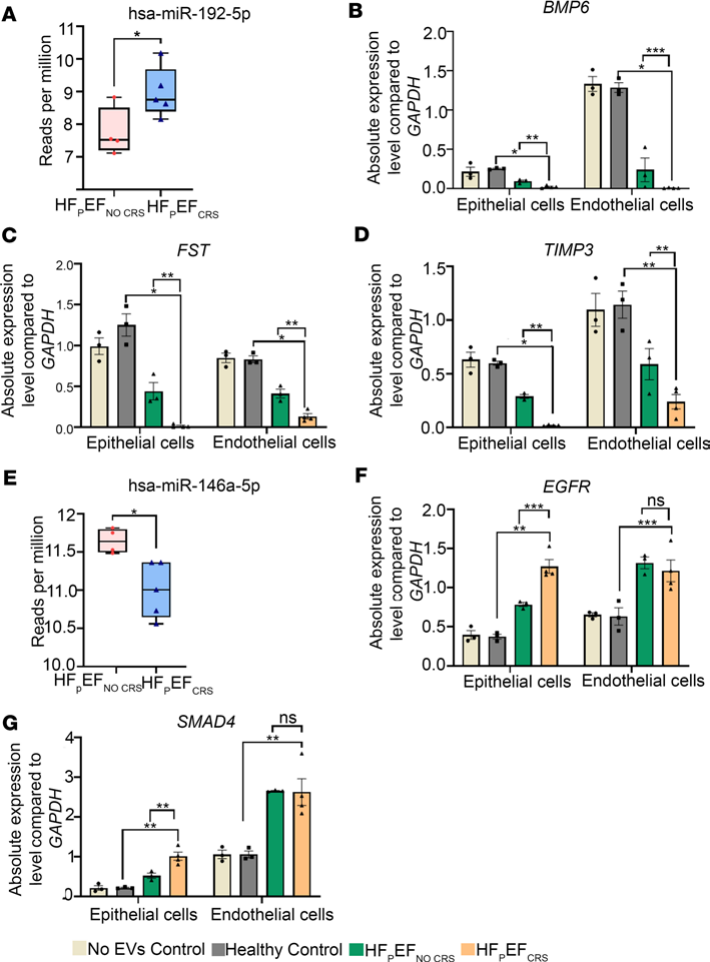
Concordant expression of the targets of hsa-miR-192-5p and miR-146a-5p in KOC. (**A**) Box-and-whisker plot showing significantly higher expression (reads per million) of hsa-miR-192-5p in HFpEF_CRS_ group compared with the HFpEF_NO CRS_. (**B**–**D**) The mRNA expression of putative miR-192-5p targets *BMP6*, *FST*, and *TIMP3* were significantly downregulated in group HFpEF_CRS_ compared with HFpEF_NO CRS_ group when analyzed by qRT-PCR. (**E**) Box-and-whisker plot showing significantly lower expression (reads per million) of hsa-miR-146a-5p in HFpEF_CRS_ group compared with the HFpEF_NO CRS_ group. (**F** and **G**) *EGFR* and *SMAD4* were significantly upregulated in the KOCs treated by EVs from HFpEF_CRS_ compared with HFpEF_NO CRS_ group in epithelial cells. *GAPDH* was used as internal loading control for all experiments. Three independent chips (technical replicates) were prepared for each biological replicate (*n* = 3 for Healthy Control and HFpEF_NO CRS_; *n* = 4 for HFpEF_CRS_) of each experimental group (averaged for each data point). Box plots represent the first quartile, median, and third quartile, with whiskers indicating minimum and maximum values. Results were analyzed by unpaired *t* test for **A** and **E** or 1-way ANOVA with Tukey’s post hoc test for **B**–**D**, **F**, and **G** and expressed as ±SEM of 3 independent experiments. *, *P* < 0.05; **, *P* < 0.01; ***, *P* < 0.001.

**Figure 9:**
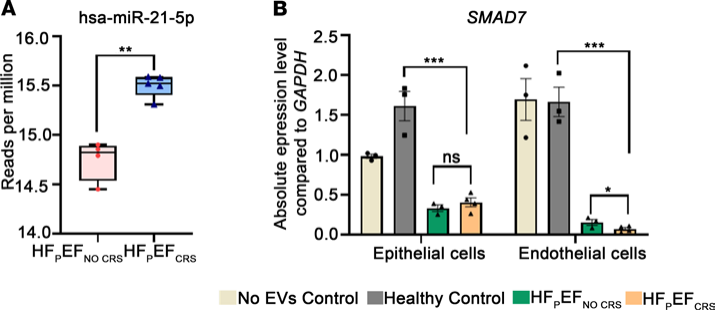
Concordant expression of the target of hsa-miR-21-5p in KOC. (**A**) Box-and-whisker plot showing significantly higher expression (reads per million) of hsa-miR-21-5p in EVs from HFpEF_CRS_ group compared with the HFpEF_NO CRS_ group. (**B**) *SMAD7* mRNA was significantly downregulated in the KOC cells treated by EVs from HFpEF_CRS_ group compared with Healthy Control group. *GAPDH* was used as internal loading control. Three independent chips (technical replicates) were prepared for each biological replicate (*n* = 3 for Healthy Control and HFpEF_NO CRS_; *n* = 4 for HFpEF_CRS_) of each experimental group (averaged for each data point). Box plots represent the first quartile, median, and third quartile, with whiskers indicating minimum and maximum values. Results were analyzed by unpaired *t* test for **A** and 1-way ANOVA with Tukey’s post hoc test for **B** and expressed as ±SEM of 3 independent experiments. *, *P* < 0.05 **, *P* < 0.01; ***, *P* < 0.001.

We sought to determine which of the 78 differentially expressed miRNAs within EVs were most strongly associated with circulating creatinine levels from all 18 study participants. We identified 15 out of 78 miRNAs that were associated with the variability of creatinine across the 18 participants through an elastic net analysis (used for prioritization of downstream targets for study, not clinical prediction). Each of these 15 miRNAs was differentially expressed between patients with or without renal dysfunction (Table 3 and Supplemental Figure 2). We annotated 1,143 high-confidence target genes of these 15 miRNAs by multiMiR and performed pathway analysis (DIANA-mirPathV.3) to identify the cellular pathways impacted. Thirty-five Kyoto Encyclopedia of Genes and Genomes (KEGG) biological processes were significantly enriched among putative mRNA targets (Figure 7 and Supplemental Table 3). Among the different annotated pathways identified, AMPK signaling (23), cell cycle (24), TGF-β signaling (25), and O-glycan biosynthesis (26) were prominent (Figure 7 and Supplemental Table 3), supporting a role for perturbation of these signaling pathways central to endothelial-mesenchymal transition/fibrosis in renal dysfunction in HFpEF. Specifically, the “TGF beta signaling pathway” (KEGG) was significantly altered in HF patients with 7 miRNAs (miR-192-5p, miR-122-5p, miR-146a-5p, miR-629-3p, miR-483-3p, miR-378c, and miR-21-5p) targeting 27 genes in the pathway. Given its biological relevance in various kidney injury models (25), we prioritized the study of the expression of TGF-β signaling pathway genes in the KOC cells treated with EVs isolated from Healthy Controls, HFpEF_CRS_, and HFpEF_NO CRS_ via qRT-PCR. *BMP6* (bone morphogenic protein 6), *FST* (follistatin), and *TIMP3* (TIMP metallopeptidase inhibitor 3) mRNA — all targets of miR-192-5p (higher in EVs from HFpEF_CRS_; Figure 8A) — were downregulated in both epithelial and endothelial cells of the chip cells treated with HFpEF_CRS_ EVs (Figure 8, B–D). Conversely, *EGFR* (epidermal growth factor receptor) and *SMAD4* (SMAD family member 4) — targets of miR-146a-5p (downregulated in HFpEF_CRS_ EVs; Figure 8E) — were upregulated in KOC cells treated with EVs from patients with HFpEF_CRS_ relative to HFpEF_NO CRS_ (Figure 8, F and G). While it was not part of the elastic net, we also investigated targets of miR-21-5p, one of the 78 dysregulated miRNAs in the HFpEF EVs, given its role in TGF-β pathway in renal dysfunction (27–29). MiR-21-5p was increased in EVs from patients with HFpEF_CRS_ (Figure 9A), and one of its targets, *SMAD7* (SMAD family member 7), was downregulated in both epithelial and endothelial cells after exposure to HFpEF_CRS_ EVs (Figure 9B).

### Experimental validation of the functional role of EV-miRNAs in CRS.

**Figure 10:**
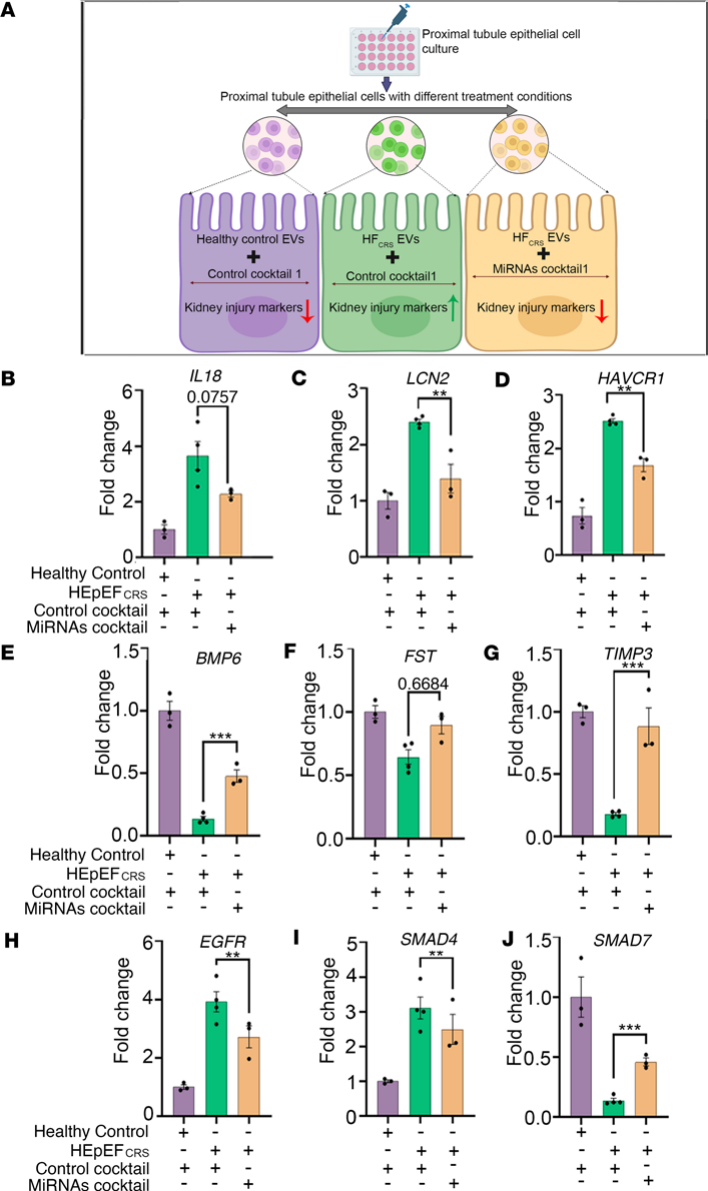
Antagonizing HFpEF_CRS_ EV–mediated miRNA effects attenuates kidney injury. (**A**) Experimental schema of miRNA cocktail 1 (comprising miRNA inhibitors of miR-192-5p and 21-5p and mimic of miR146a-5p) designed to antagonize the effects of key CRS cargo miRNAs on recipient cells (created with BioRender.com). (**B**–**D**) Amelioration of all 3 kidney injury markers (*IL18*, *LCN2*, *HAVCR1*) in the “HFpEF_CRS_+MiRNAs cocktail 1 treated group” compared with “HFpEF_CRS_+Control cocktail 1 treated group.” (**E**–**J**) QRT-PCR analyses showed marked upregulation of *BMP6*, *FST*, *TIMP3*, and *SMAD7* and significant downregulation of *EGFR* and *SMAD4* in the “HFpEF_CRS_+MiRNAs cocktail 1 treated group” compared with “HFpEF_CRS_+Control cocktail 1 treated group.” *GAPDH* was used as internal loading control. *n* = 3 for Healthy Control+Control cocktail 1 treated group; *n* = 4 for HFpEF_CRS_+Control cocktail 1 treated group; *n* = 3 for HFpEF_CRS_+miRNAs cocktail 1 treated group. Results were analyzed by unpaired *t* test and expressed as ±SEM of 3 independent experiments. **, *P* < 0.01; ***, *P* < 0.001.

To experimentally validate the functional role of the EV-miRNAs in mediating renal injury and regulating the TGF-β pathway, we designed 2 sets of miRNA inhibitor/mimic combinations to antagonize or reproduce the biological effects of the HFpEF_CRS_ EV-miRNAs in renal epithelial cells. The combination of miRNA inhibitors for miR-192-5p and miR-21-5p (both upregulated in HFpEF_CRS_ EVs) and the mimic for miR-146a-5p (downregulated in the HFpEF_CRS_ EVs) would be expected to antagonize the effects of HFpEF_CRS_ EV-miRNAs when transfected into the renal epithelial cells (Figure 10A). This combination, referred to as miRNAs cocktail 1, ameliorated the expression of kidney injury markers (*IL18*, *LCN2*, and *HAVCR1*; Figure 10, B–D), as well as *CST3* gene expression (Supplemental Figure 3A), and markedly upregulated the expression of *BMP6*, *FST*, *TIMP3*, and *SMAD7* while downregulating the expression of *EGFR* and *SMAD4* (Figure 10, E–J) when compared with a control cocktail 1 (consisting of the scrambled versions of the inhibitors and mimic) in renal epithelial cells exposed to HFpEF_CRS_ EVs. Thus, transfection of miRNAs cocktail 1 into the EV recipient renal epithelial cells appeared to mitigate the effect of the HFpEF_CRS_ EVs, mitigating renal injury markers and restoring mRNA target genes toward the baseline control levels (cells transfected with control cocktail 1 and exposed to Healthy Control EVs).

**Figure 11:**
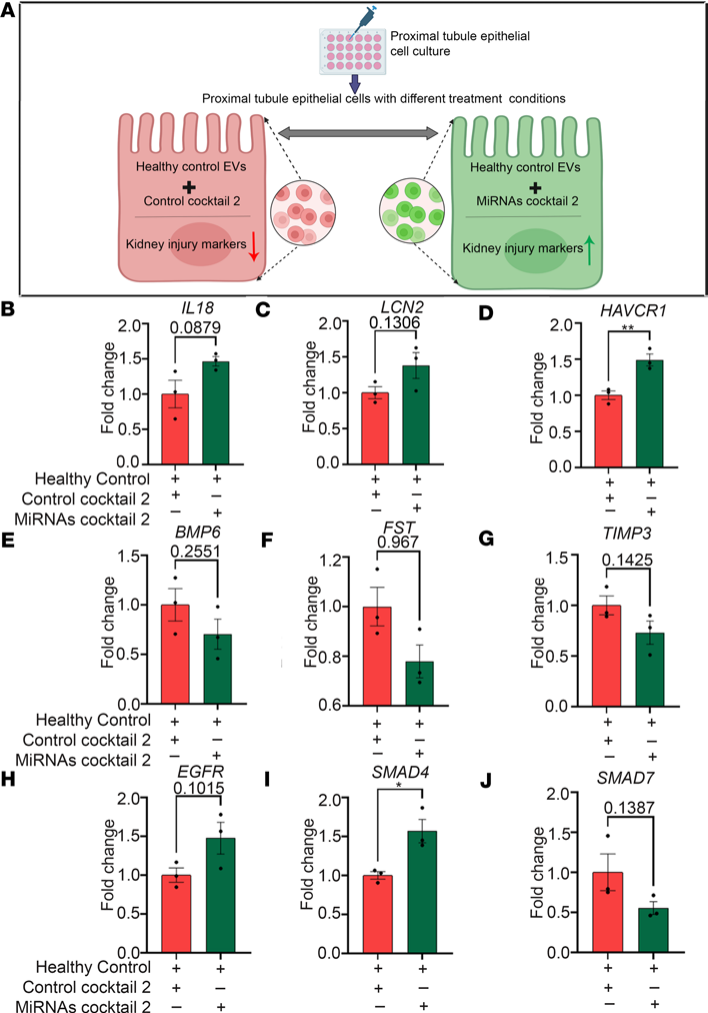
MiRNA cocktail 2 mimics the effects the HFpEF_CRS_ on renal epithelial cells. (**A**) Experimental schema of miRNA cocktail 2 comprising mimics of miR-192-5p and miR-21-5p and miRNA inhibitor of 146a-5p designed to mimic the effects of CRS EVs on recipient renal epithelial cells (created with BioRender.com). (**B**–**D**) mRNA expression of kidney injury markers (*IL18*, *LCN2*, *HAVCR1*) markedly upregulated in the “Healthy Control EVs+MiRNAs cocktail 2 treated group” compared with “Healthy Controls+Control cocktail 2 treated group.” (**E**–**J**) QRT-PCR analyses showed marked downregulation of *BMP6*, *FST*, *TIMP3*, and *SMAD7* and marked upregulation of *EGFR* and *SMAD4* in the “Healthy Control+miRNAs cocktail 2 treated group” compared with “Healthy Control+Control cocktail 2 treated group.” *GAPDH* was used as internal loading control. *n* = 3 replicates for each group. Results were analyzed by unpaired *t* test and expressed as ±SEM of 3 independent experiments. *, *P* < 0.05; **, *P* < 0.01.

Conversely, the combination of the mimics for miR-192-5p and miR-21-5p (both upregulated in HFpEF_CRS_ EVs) and the inhibitor for miR-146a-5p (downregulated in HFpEF_CRS_ EVs), referred to as miRNAs cocktail 2, was designed to mimic the effect of HFpEF_CRS_ EVs when transfected into recipient renal epithelial cells (Figure 11A). Indeed, miRNAs cocktail 2 increased the expression of kidney injury markers (*IL18*, *LCN2*, and *HAVCR1*) (Figure 11, B–D) along with *CST3* gene (Supplemental Figure 3B), while downregulating the expression of *BMP6*, *FST*, *TIMP3*, and *SMAD7* and increasing the expression of *EGFR* and *SMAD4* (Figure 11, E–J) in renal epithelial cells exposed to Healthy Control EVs (when compared with control cocktail 2–transfected, consisting of the scrambled versions of the inhibitor and mimics, cells). This pattern of expression was in concordance with exposure to HFpEF_CRS_ EVs, suggesting that the effects of these EVs on renal epithelial cells were largely mediated by their cargo miRNAs.

While there is no existing data set of human renal tissue mRNA expression for patients with CRS, we analyzed human tissue in renal transplant patients with or without acute kidney injury undergoing biopsy to determine whether similar cellular pathways identified in our in silico model were dysregulated in vivo (Gene Expression Omnibus [GEO] data set accession GSE30718). We identified 736 significantly differentially expressed genes between patients with or without kidney injury (at an FDR 5%) out of a total 20,848 genes in these samples (Supplemental Figure 4A; enriched KEGG pathways in Supplemental Figure 5 and Supplemental Table 4). Interestingly, 1,094 out of 1,143 mRNAs that were putative targets of the 15 EV-miRNAs associated with plasma creatinine overlapped with the 20,848 genes detected in the microarray. Of these 1,094 miRNA target genes, 74 genes were present within the 736 differentially expressed genes between kidney tissue with or without injury, representing significant enrichment in this data set (Fisher *P* < 0.001). Notably, 4 of these genes were related to the TGF-β pathway (Supplemental Figure 4B).

### Expression quantitative trait loci result.

Mendelian randomization was used to determine whether genetically determined levels of mRNA expression of the candidate genes were associated with kidney function. There were 2 genes with 1 or more expression quantitative trait loci (eQTL) that were significantly associated with estimated glomerular filtration rate (eGFR): FST and SMAD7 (Supplemental Table 5). FST demonstrated a significant correlation with eGFR (0.005 [95% CI: 0.002–0.007] mL/min/1.73 m^2^ increase in eGFR per standardized unit change in mRNA expression, *P* = 5.5 × 10^–5^), after adjusting for multiple testing, consistent with a protective effect of higher FST expression on renal function. This was consistent with our observation of downregulation of FST in the KOC treated with HFpEF_CRS_.

## Discussion

Cardiac and renal diseases are influenced by synergistic systemic factors (30) and frequently occur concurrently, with amplified clinical consequences for individuals with both. In this context, efforts to resolve how worsening cardiac function influences renal dysfunction are critical to mitigate joint consequences. Studies in CRS have focused on the role of renal hemodynamics, uremia, and accompanying metabolic changes as prime drivers of renal/cardiac signaling that reinforces myocardial alterations (31), with a broad assessment that overall inflammatory and other uremic toxins during kidney injury lie at the heart of poor cardiac prognosis in HF. This focus on systemic inflammatory and other signaling moieties independent of hemodynamic may be particularly pertinent to HFpEF, where mechanisms of CRS remain poorly understood. While certain shared inflammatory and metabolic stimuli can elicit both renal and cardiac dysfunction in CRS, the potential nature of EV signaling has been less well studied.

In this study, we leverage the use of a human KOC to study the functional role of patient-derived EVs in CRS associated with HFpEF. The ability to use human model systems to study patient-derived materials to ultimately derive clinically relevant results that can be translated back to human studies is of particular importance in this study given the known shortcomings of previous animal and cell culture models. Our study suggests that EVs from HFpEF patients with CRS are directly injurious to renal epithelial cells in the short term, driving expression of injury markers (*LCN2*, *IL18*, *HAVCR1*) that have previously been shown to be elevated in the urine of patients with CRS (32, 33). Furthermore, these EVs regulate transcriptional pathways that may drive epithelial-mesenchymal transition and renal fibrosis. Notably, our findings focus on the TGF-β signaling pathway and suggest a broad role for this pathway in renal dysfunction/injury in humans. Moreover, genetic alterations in key members of this pathway may predispose to kidney disease, suggesting a broader role for this pathway in kidney disease.

The fundamental finding here is the application of a microfluidic technology (KOC) as a model of renal proximal tubular physiology to characterize EV-based mechanisms of renal injury in HF that begin at the renal-extrarenal interface (circulation). While the concept of an in vitro chip-based system to test renal injury has been previously advanced (34–37), its application to evaluate the importance of transorgan communication in renal injury via EVs has not been previously described to our knowledge. Careful profiling of the EV long RNA transcriptome allowed the identification (using deconvolution approaches) of putative source organs of circulating EVs in HFpEF. Not surprisingly, multiple organs (including the heart, immune cells, adipose tissues) contribute to the circulating EV populations, consistent with HFpEF being a multisystem disease. The characterization of the EV small RNA transcriptome coupled with the use of curated databases of miRNA-mRNA targets allowed us to identify several mRNA targets of differentially abundant miRNAs (between individuals with and without CRS) that are part of the TGF-β signaling network and demonstrated that these specific mRNAs were altered in renal tubular cells on the KOC. The TGF-β pathway has been associated with the development of a wide array of kidney diseases and may regulate both miRNA-mediated renal injury (38) and the expression of *CST3* (cystatin C gene), which is positively controlled by 2 transcription factors: IRF-8 and PU.1 (39, 40), which are both activated by TGF-β/SMAD4 (41, 42), while IRF-8 is negatively regulated by SMAD7.

More specifically, miR-192-5p and miR-21-5p expression was enhanced in individuals with HF and CRS, with a corresponding downregulation of key targets previously implicated as protective in renal fibrosis — e.g., miR-192-5p: *BMP6* (43, 44), *TIMP3* (45–47); miR-21-5p: *SMAD7* (48–50). Previously, it was noticed that miR-192-5p could promote ischemia/reperfusion-induced renal injury in rats (51). Previous reports have also revealed that miR-192-5p directs TGF-β–mediated collagen deposition during diabetic renal injury via interacting with SMAD-interacting protein 1. MiR-21-5p has been considered to play a variety role in regulation of different kidney diseases like allograft dysfunction (52) and diabetic nephropathy (53).

The perturbation of the mRNA targets of these miRNAs in the pathogenesis of renal disease has been previously demonstrated. Indeed, deletion of BMP6 aggravates renal injury and fibrosis by inducing inflammatory cells in renal proximal tubule cells (43). Also, it was observed that administration of FST as an antagonist of activin can reduce fibrosis during unilateral ureteral obstruction in a preclinical model (54). In line with this, other studies showed that deletion of TIMP3 leads to increased interstitial fibrosis, as well as higher synthesis and deposition of collagen I, suggesting activation of fibroblasts (46). Absence of TIMP3 results in renal injury in murine models (47). In addition to these studies, SMAD7 is shown to play a protective role against a wide range of renal pathology, and deletion of SMAD7 leads to diabetic kidney injury (48). SMAD7 also protects from acute renal injury by releasing tubular epithelial cell cycle arrest at the G_1_ stage during ischemia/reperfusion-induced renal injury in vivo (49). Additionally, disruption of SMAD7 results in angiotensin II–mediated hypertensive nephropathy (50). MiR-146a-5p, a known negative regulator of the TGF-β pathway (55, 56), was downregulated in plasma of individuals with HF and CRS. Fibrosis-enhancing gene targets of miR-146a-5p were increased in renal tubular cells on a chip (*EGFR*, *SMAD4*). SMAD4 plays a key role in regulating TGF-β–induced collagen expression and promotes SMAD3-mediated renal fibrosis (57) while activation of EGFR serves as prognostic biomarker during chronic kidney disease (58). Finally, as *CST3* is controlled by the TGF-β pathway, it is likely that EVs coming from HFpEF patients with CRS may trigger the upstream cascades converging on IRF-8 and PU.1 transcriptional activity and subsequently increase the expression of cystatin C.

Critically, our initial observations were experimentally validated using gain-of-function/loss-of-function approaches using cocktails of miRNA mimics and miRNA inhibitors transfected into the putative EV recipient cells to either oppose or reproduce the effects of the circulating EVs. These experiments supported our initial observations that the cargo miRNAs (miR-192-5p, miR-21-5p, and miR-146a-5p) that are more abundant in the HFpEF_CRS_ EVs indeed regulate the TGF-β/SMAD pathway and the renal injury patterns initially noted. These observations also support future development of therapeutics targeting these miRNAs and pathways.

The ability to simultaneously profile circulating EV cargo and to determine the functional implications in the target organ of interest (the kidney) further establishes EVs and their cargo as relevant functional biomarkers of CRS. Prior work studying EVs as biomarkers and mediators of kidney diseases spans a breadth of conditions: e.g., glomerulonephritis (59, 60), acute and chronic kidney disease (61–64), and posttransplant rejection and homeostasis (65, 66), among others. A consistent finding across studies has been the utility of specific molecular mediators, including miRNAs within urinary or circulating EVs, as early biomarkers for kidney diseases in both preclinical models of (67) and in human diabetic nephropathy (68), with potential implications on fibrosis and immune mechanisms: e.g., miR-320c (68, 69), miR-29c (70), and miR-19b (71). Our findings here align with and extend beyond these results by not only identifying EV contents but also establishing their functional role in renal injury, fibrosis, and dysfunction. However, a potential confounder in our study was that individuals with HF and CRS had poorer renal function at study entry, which may reflect prevalent renal dysfunction as seen in type 2 CRS. Whether the circulating EVs from these individuals represent a profile of prevalent kidney injury in the setting of chronic HFpEF (type 2 CRS) or propensity to progressive injury with therapy for HF (type 1 CRS) remains open. Moreover, whether this can be generalized to all forms of HF (e.g., including HF with reduced ejection fraction) and to other comorbid conditions known to influence circulating EV profiles and renal disease (e.g., diabetes, obesity, hypertension) is an area of active interest. Finally, it is important to note that our study was not designed to qualify these EV-miRNAs as clinical biomarkers, but rather to demonstrate the utility of a human KOC platform for biomarker and pathway discovery. Undoubtedly, measurement of these EV-miRNAs in independent cohorts will be an important step in validation of these potential biomarkers.

This study represents a first step toward use of in silico technology to permit isolation of EV effects on physiology in a unique clinical context (CRS). Nevertheless, there are important limitations in our approach. It is well accepted that there are a diverse number of extracellular particles in biofluids, including ribonuclear protein (RNP) complexes, lipoproteins, exomeres, and supermeres and that any isolation method for EVs may coisolate these other particles. There also remains some controversy about the carriers for miRNAs in plasma, including their association with RNPs. To increase the rigor of our study, we used 2 complementary isolation methodologies to increase our confidence that EVs may indeed be the functional entity in our study, and EVs used in our studies were treated with RNase (Thermo Fisher Scientific) to degrade RNA molecules not protected within EVs. Nonetheless, it remains possible that entities other than EVs may also carry the bioactive miRNAs and mediate some of these effects. Secondly, while our experimental validation studies point to the miRNA cargos of these biologically active EVs as important mediators of their effect, other contents of these EVs, including proteins, lipids, or metabolites, may have synergistic or opposing effects. Additionally, whether the effects of the HFpEF_CRS_ EVs on renal injury in the KOC model correlates with urinary expression of these markers in the patients from whom the EVs were derived would be interesting to study in the future.

The relevance of EV uptake into the appropriate cell types, especially in vivo, still needs to be elucidated. While our data suggest direct uptake of labeled plasma EVs into endothelial and epithelial cells of the KOC, lipophilic dyes like Dil, widely used for EVs staining, could also generate false-positive EV signals due to dye aggregation. Future imaging methods (such as more specific targeted dyes) might be used to enable accurate, long-term imaging of EVs for preclinical and clinical settings. As previously pointed out, the lack of validated murine models of CRS, and especially HFpEF-associated CRS, a focus of our study, directed our efforts to use the human KOC as a more relevant model to investigate. Future development of these models, as well as standardized approaches to administer and assess biodistribution of EVs in vivo, may facilitate more mechanistic, physiologically relevant preclinical studies. Certainly, a deeper transcriptional approach with more diverse cell types (e.g., renovascular cells and pericytes) will be critical to model a complex renal cellular ecosystem and broadly cover potential mechanisms of renal injury (e.g., by use of single nuclear RNA-Seq in more complex chip systems).

**Figure 12:**
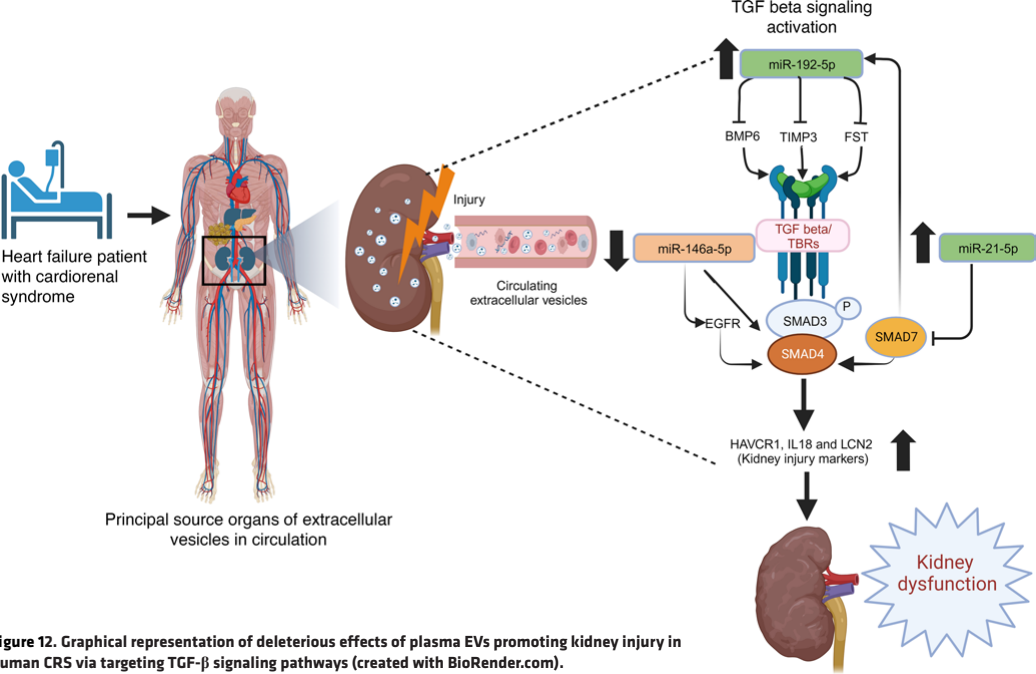
Graphical representation of deleterious effects of plasma EVs promoting kidney injury in human CRS via targeting TGF-β signaling pathways (created with BioRender.com).

In summary, we leveraged a human KOC to decipher the possible contribution of circulating EVs and their RNA cargos in mediating CRS in patients with HFpEF. Our system demonstrated the injurious effect of these EVs along with their sources and contents on renal epithelial and endothelial cells and identified key signaling pathways related to TGF-β that may be targeted by miRNAs contained within these EVs (Figure 12). Notably, these data add to previous data from animal models that also implicate these pathways in renal injury and are corroborated by complementary human data that suggest an important role for this pathway in renal disease.

## Methods

Further information can be found in Supplemental Methods.

### Study population and plasma collection.

A total of 12 patients with HFpEF with or without CRS were consented under an approved Institutional Review Board (IRB) protocol (2016P001250), as part of the Circulating RNAs in Acute Heart Failure (CRUCiAL, NCT03345446). HFpEF with CRS (HFpEF_CRS_; 6 patients were used for c-DGUC, and out of 6 patients, 4 were again used for SEC) and without CRS (HFpEF_NO CRS_: 6 patients were used for c-DGUC, and out of 6 patients, 3 were again used for SEC) were used for the studies. For this study, CRS was defined as present in those individuals with HF and renal dysfunction. Following the Acute Dialysis Quality Initiative (ADQI) criteria (7), Kidney Disease: Improving Global Outcomes (KDIGO) (72), and 7th ADQI Consensus Conference for Definition and Classification of Cardiorenal Syndrome (73), CRS was defined as an increase in creatinine of at least 0.3 mg/dL following admission for acute decompensated HF (type 1 CRS) or an estimated glomerular filtration rate (eGFR) less than 60 mL/min/1.73 m^2^ in the presence of diagnosed HF (type 2 CRS). Peripheral venous blood was collected at hospital admission and processed within 60 minutes of venipuncture via centrifugation (500*g* for 10 minutes at room temperature). The supernatant was recentrifuged at 2,500*g* for 10 minutes. Plasma samples were stored at –80°C until EV isolation. Plasma from 6 samples for c-DGUC and 3 samples for SEC were included as control participants (Healthy Control) were collected following the same protocol.

### Isolation of EVs.

Plasma samples were processed following either c-DGUC– or SEC-based Izon technology (Izon Science) for EV isolation, as previously described (21, 74). Fractions 6–10 for c-DGUC and 7–10 for SEC methods were pooled and used for all downstream experiments as optimized by our group. The plasma concentration of the EVs in the samples (pooled) was 1.98 × 10^11^ EV particles/mL as measured by Spectradyne. Following our isolation from 0.5 mL of plasma, we are left with a concentration of 1.8 × 10^10^ (on average) for the samples, eluted into 1 mL.

### Western blot for EVs isolated through c-DGUC and SEC.

Western blot analysis was done as described (21). Concentrated EV suspensions from plasma were lysed for protein extraction (RIPA lysis buffer; 1× protease and phosphatase inhibitor cocktail, Thermo Fisher Scientific) for 20 minutes at 4°C. Protein concentration was quantified with Pierce BCA Protein Assay Kit (Thermo Fisher Scientific) followed by SDS-PAGE. Gels were transferred to PVDF membranes (MilliporeSigma) and blocked with 5% bovine serum albumin (MilliporeSigma) for 1 hour at room temperature. Primary antibodies against CD81, CD63, Alix, Syntenin, and 58K Golgi protein were incubated at 4°C overnight at 1:1,000 concentration followed by incubation with secondary HRP-antibodies (Supplemental Table 5) for 1 hour at room temperature. Blots were developed using the Super Signal Femto developer (Thermo Fisher Scientific).

### In vitro renal models (KOC and proximal tubule epithelial cell culture in well).

The goal of the KOC technology is to simulate the microenvironment in the proximal nephron, including exposure of the renal environment to circulating plasma (endothelial interface) and the functional response of the renal epithelium to these contents (epithelial surface). By design, each chip includes epithelial cells in the apical channel and endothelial cells in the basal channel. These 2 channels are parted by a porous membrane which allows the cell-to-cell interaction mimicking the in vivo system.

To construct the human Proximal Tubule Kidney Chip, polydimethylsiloxane chips (Chip-S1; Emulate) were used, and the culture was set up following the manufacturer’s protocol. Briefly, the bottom channel was seeded with Human Renal Microvascular Endothelial Cell (2 × 10^6^ cells/mL; Cell Systems), and on the next day the top channel was seeded with Human Renal Proximal Tubule Epithelial Cell (1.0 × 10^6^ cells/mL, hRPTEC; Lonza). Chips were maintained for another 96 hours at this condition before EV experiments.

Remaining hRPTECs were seeded to a final cell density of 1.6 × 10^5^ cells/mL in complete maintenance medium. Then 500 μL of cell suspension was added to each well of 24-well plates. Cells were maintained undisturbed and allowed to fully attach until the next day, followed by maintenance for another 96 hours prior to EV treatment.

### Application of human-derived EVs to the KOC and proximal tubular epithelial cells.

EV particles isolated from plasma (see above) at a final concentration of 1.8 × 10^10^ EVs/mL (in 1 mL) were perfused for 72 hours onto a total of 3 × 10^6^ cells, which translates to 6,000 EVs/cell over 72 hours. Successful EV dosing resulted in significantly higher uptake of EVs to the bottom channel compared with top channel. For the epithelial cell culture cells were directly treated with EVs at the same concentration as above.

To determine the successful EV uptake within the chip, purified EVs from Healthy Control participants were labeled with a tracer dye, Dil (5 mmol/L, Thermo Fisher Scientific) for 20 minutes at 37°C. To get rid of the excess dye, EVs were centrifuged at 750*g* for 2 minutes at 37°C using a spin column (Exosome Spin Columns, MW 3,000, Thermo Fisher Scientific) and resuspended in 1× PBS. This was repeated twice. Dil-stained EVs were diluted at 1:50 into degassed complete endothelial media and added as a single bolus to each bottom inlet (endothelial surface) after aspirating all media from both inlets and outlets followed by uninterrupted flow for 3 days. “No EVs Control” group was exposed to PBS alone.

### Effluent sampling from the chips.

Effluents were collected from all Pod outlet reservoirs. The amount of cystatin C in the sample effluents of different groups (HFpEF_CRS_, HFpEF_NO CRS_, Healthy Control, and No EVs Control) was quantified via ELISA (Abcam) following manufacturer protocol (expressed as pg cystatin C/mL cellular effluent).

### RNA extraction and quantification.

After 3 days of dosing chips with human-derived EVs, the chips were disconnected, washed with 1× PBS, and filled with RNAlater (Invitrogen) to preserve cells for RNA extraction. Similarly, epithelial cells from 24 wells were also maintained in RNAlater. The PureLink RNA Mini Kit (Thermo Fisher Scientific) was used following the manufacturer’s protocol. Total RNA was eluted in 20 μL, treated with DNase, and “cleaned up” using RNA Clean & Concentrator-5 with DNase I (Zymo Research) following manufacturer’s protocol. Final RNA concentration was quantified by spectrophotometry (NanoDrop 2000, Thermo Fisher Scientific).

The High-Capacity cDNA Reverse Transcription Kit (Thermo Fisher Scientific) was used for cDNA synthesis from RNA. For amplification and quantification of selected genes (*IL18*, *LCN2*, *HAVCR1*, *BMP6*, *FST*, *TIMP3*, *EGFR*, *SMAD4*, *SMAD7*, *CST3*, and *GAPDH*), the ExiLENT SYBR Green master mix (Exiqon) was used on a QuantStudio 6 Flex Real-Time PCR System up to 40 amplification cycles. Any amplification cycle (Ct) greater than or equal to 40 was assigned as a “negative threshold” and was therefore not included in our calculations. For all the kidney chip experiments, absolute gene expression was quantified by 2^-Δ^Ct method after normalization of genes of interest to the internal control *GAPDH* whereas relative gene expression was used for the conventional cell cultures. All qRT-PCR primer sequences are summarized in Supplemental Table 2.

### Small RNA-Seq of plasma EV samples.

We performed small RNA-Seq on the extracellular RNA isolated from 1 mL of plasma of patients with HFpEF or Healthy Controls to identify the differences in extracellular RNA cargo, following the previously published protocol with some modifications (75). Plasma extracellular RNA was isolated using the miRNeasy Serum/Plasma Midi Kit (Invitrogen), and libraries were constructed using the NEBNext Small RNA Library Prep Set for Illumina (New England Biolabs). Size selection of libraries was performed by gel electrophoresis with excision of the 140 to 160 nucleotide bands (corresponding to 21- to 40-nucleotide RNA fragments) and sequenced on an Illumina HiSeq 2000.

Bioinformatic processing was performed using TIGER, as described (76). Briefly, Cutadapt (v2.10) (77) was used to trim 3′ adapters for raw reads. All reads with fewer than 16 nucleotides were designated as “too short” and discarded. Quality control on both raw reads and adaptor-trimmed reads was performed using FastQC (v0.11.9) (78). The adaptor-trimmed reads were mapped to the GENCODE GRCh38.p13 genome, in addition to rRNA and tRNA reference sequences by Bowtie (v1.3.0) (79), allowing only 1 mismatch. Significantly differentially expressed miRNAs between HFpEF and Healthy Control samples with absolute fold-change ≥ 1.5 and FDR-adjusted *P* ≤ 0.05 were detected by DESeq2 (v1.30.1) (80) using total host small RNA reads as normalization factor.

### Identification of miRNAs associated with creatinine.

We used elastic net regression to select a group of miRNAs associated with circulating creatinine levels. Log-transformed reads per million expression of the significantly differentially expressed miRNAs was included as features in an elastic net regression for creatinine level (in mg/dL) as the response variable (glmnet in R). The miRNAs selected by elastic net were used for target gene annotation using the multiMiR package. Only the genes annotated as target genes in at least 2 out of 3 databases, including mirecords, mirtarbase, and tarbase, were retained as high-confidence target genes.

### Human renal transplant biopsy samples.

To determine if the mRNA targets of the EV-miRNAs associated with creatinine (by elastic net) in HFpEF were deregulated in human kidney tissue, we queried a published microarray data set of renal transplant patients with acute kidney injury (GEO GSE30718, ref. 81). From this study, 28 individuals with acute kidney injury (“sample” group) and 11 “pristine” protocol biopsy samples (control group) of the microarray data set (GSE30718) were analyzed based on GEO2R R script with following modifications: (i) the probes without gene symbol annotation and probes mapped to multiple gene symbols were discarded before differential expression analysis, and (ii) for gene symbols mapped by multiple probes, the probe with the smallest *P* value was kept as the representative. Significantly differentially expressed genes with absolute fold-change ≥ 1.5 and FDR-adjusted *P* value ≤ 0.05 were detected by linear model using the limma package. The differentially expressed genes were used in KEGG pathway overrepresentation analysis by WebGestalt R package. Fisher exact test was used to test the enrichment of the differentially expressed genes in miRNA target genes compared with all microarray genes.

### EQTL analysis.

SNPs associated with mRNA levels for each of the 6 candidate genes (*BMP6*, *EGFR*, *FST*, *SMAD4*, *SMAD7*, *TIMP3*) were identified using data from the GTEx version 8 resource (82). The best performing gene expression model for each gene was identified by selecting the model with the highest performance *r*^2^, comparing PrediXcan, UTMOST, and JTI methods for gene expression imputation (83–85). The SNPs identified by the best-performing model were then used in the downstream eQTL analysis. The association between each of these eQTL and kidney function was examined using GWAS summary statistics of eGFR (*n* = 1,201,909) (86), and those genes with 1 or more SNPs associated with eGFR levels at genome-wide significance (*P* < 5 × 10^–8^) were taken forward for genetic association analysis. For the selected genes, a linkage disequilibrium–reduced (*R*^2^ = 0.05) set of eQTL was selected using PLINK v1.90. An inverse variance weighted meta-analysis approach was used to test the association between predicted gene levels (exposure) and eGFR levels (outcome) using the Mendelian Randomization R package (87). A Bonferroni-corrected association *P* < (0.05/5 genes = 0.01) was considered significant.

### Statistics.

Values for Figures 4, 7, 8, 9, 10, and 11 and Supplemental Figure 3 were presented as means ± SEM of 3 independent experiments; data were analyzed by GraphPad Prism (Version 9.3.1); and statistical significance was assessed by an unpaired 2-tailed *t* test between 2 means, or 1-way ANOVA was used to assess differences among multiple groups, followed by Tukey’s post hoc test. Results with a *P* < 0.05 were considered significant.

### Study approval.

The study was approved by the IRB at Mass General Brigham, and written informed consent was received prior to participation in the study. The trial is registered in ClinicalTrials.gov as NCT03345446, Circulating RNAs in Acute Congestive Heart Failure (CRUCiAL).

### Data availability.

All RNA-Seq data have been deposited at NCBI dbGaP (accession number phs003403.v1.p1).Values for all other data points in graphs are reported in the Supporting Data Values file.

## Author contributions

EC, RSR, and VK designed and carried out experiments on the KOC with EC leading the study to completion. GPDO, GL, and PG conducted selected experiments. ESL and JEH collected and provided clinical samples and clinical metadata as well as provided analysis related to Figure 6. M Spanos and HIL assisted in data analysis related to patient data. M Shi, TWMF, and JDM conducted the Mendelian randomization analysis. QS did the statistical and computational analysis. ICG and JL provided critical review of the manuscript. KK helped design KOC experiments. EC, RR, M Spanos, RS, and SD participated in writing the manuscript. RS and SD were responsible for supervision of data analysis and for the final manuscript. SD was responsible for overall supervision of the experimental design and funding for the project (and is therefore the last author listed).

## Supplementary Material

Supplemental data

Supporting data values

## Figures and Tables

**Table 3:**
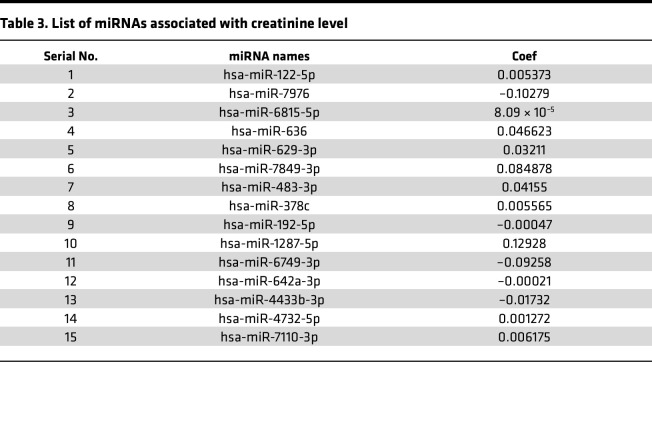
List of miRNAs associated with creatinine level

**Table 1:**
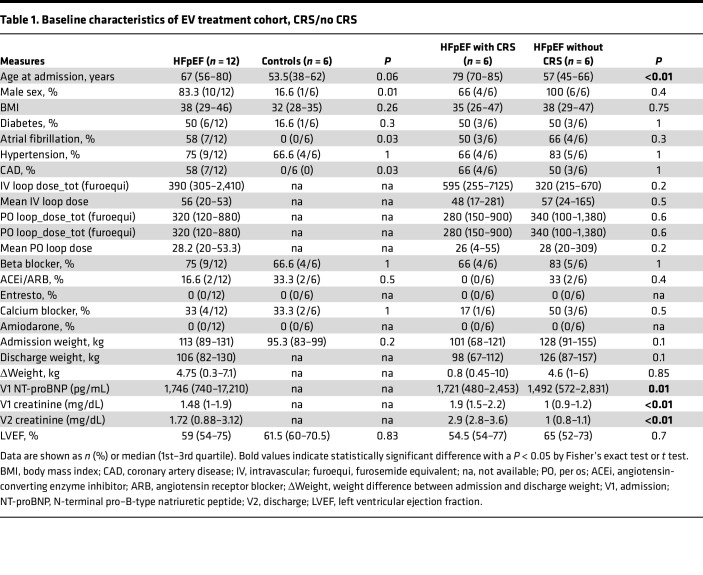
Baseline characteristics of EV treatment cohort, CRS/no CRS

**Table 2:**
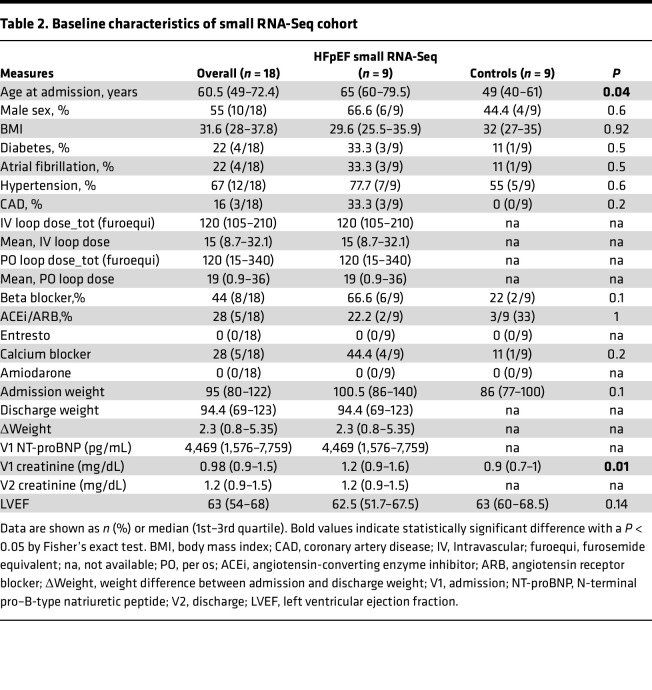
Baseline characteristics of small RNA-Seq cohort

## Version 1 09/14/2023

In-Press Preview

## Version 2 11/22/2023

Electronic publication
