# Supplementary material for: Circulating extracellular vesicles in human cardiorenal syndrome promote renal injury in a kidney-on-chip system
Source: JCI Insight. 2023 Nov 22;8(22):e165172. doi: 10.1172/jci.insight.165172 (PMC10721327; doi:10.1172/jci.insight.165172)
Supplement: Supplemental data [file jciinsight-8-165172-s268.pdf]

Supplemental Table 1: **Baseline characteristics of long RNA sequencing cohort**

Measures	Overall (18)	Cr Low (9)	Cr High (9)	p-value
Age at admission, Years	69 (64-75)	68 (68-72)	74 (70-77)	0.04
Male Sex, %	28 (5/18)	33 (3/9)	22 (2/9)	0.5
Creatinine (mg/dL)	1(0.8-1.3)	0.8(0.7-0.9)	1.22(1.1-1.6)	**<0.01**
Diabetes, %	22 (4/18)	44 (4/9)	0 (0/9)	0.01
EGFR, ml/min/1.73m2	69(51-89)	85(70-94)	49(28-58)	<0.01

Median (1st,3d quartile)
**Fisher's exact test*

Supplemental Table 2: Primers list

Sl.No.	Gene Names	Sequences (5 ⟶ 3)
1.	*LCN2* F	CCA CCT CAG ACC TGA TCC CA
	LCN2 R	CCC CTG GAA TTG GTT GTC CTG
2.	*IL18* F	TCT TCA TTG ACC AAG GAA ATC CG
	IL18 R	TCC GGG GTG CAT TAT CTC TAC
3.	*HAVCR1*F	TGGCACTGTGACATCCTCAGA
	*HAVCR1*R	GCAACGGACATGCCAACATA
4.	*TIMP3* F	TTT GCC CTT CTC CTC CAA TAC
	TIMP3 R	TCT GCT TGT TGC CTT TGA
5.	*FST* F	GCC TAT GAG GGA AAG TGT ATC AA
	FST R	CCC AAC CTT GAA ATC CCA TAA AC
6.	*BMP6* F	GCT CTC CAG TGC TTC AGA TTA C
	BMP6 R	CAC ATA CAG CTA ATG CTT CCT
7.	*SMAD7* F	GAA ATC CAA GCA CCA CCA AAC
	SMAD7 R	CAC ACT CAC ACT CAC ACA CA
8.	*EGFR* F	AGT AAC AAG CTC ACG GAG TT
	EGFR R	CAA GGA CCA CCT CAC AGT TAT TT
9	*CST3* F	CCTTCCATGACCAGCCACATCT
	CST3 R	AGG CGT CCT GAC AGG TGG ATT T
10.	*GAPDH* F	GGA GCG AGA TCC CAA AAT
	GAPDH R	GGC TGT TGT CAT ACT TCT CAT GG

Supplemental Table 3: DIANA mirPath v.3 KEGG comparative pathway analysis

	p-value	#genes	#miRNAs
Prion diseases	1.26E-13	9	4
Lysine degradation	9.41E-07	18	4
Proteoglycans in cancer	1.52E-06	61	6
AMPK signaling pathway	2.37E-06	49	5
Chronic myeloid leukemia	1.46E-05	29	6
Cell cycle	4.08E-05	45	6
TGF beta signaling pathway	5.82E-05	27	6
Signaling pathways regulating pluripotency of stem cells	8.06E-05	46	5
Other types of O-glycan biosynthesis	0.000242	11	4

P-value threshold: 0.001

FDR correction

30

Supplemental Table 4:

KEGG pathways for GSE30718 dataset

Measures	Overall (18)	HFpEF (12)	Controls (6)
Complement and coagulation cascades	4.280836032	6.29E-07	2.05E-04
Pathogenic Escherichia coli infection	4.611627907	7.22E-06	0.001177138
Pathways in cancer	1.895842579	1.21E-05	0.001320098
Mineral absorption	4.558880529	1.96E-05	0.001419735
Regulation of actin cytoskeleton	2.480824872	2.18E-05	0.001419735
Adherens junction	3.816335594	2.66E-05	0.001447672
Phagosome	2.503021726	2.79E-04	0.011653853
PI3K-Akt signaling pathway	1.91065563	2.86E-04	0.011653853
Focal adhesion	2.230498422	4.42E-04	0.016027348
Bacterial invasion of epithelial cells	3.141931175	6.49E-04	0.021144503
Leukocyte transendothelial migration	2.642078488	7.65E-04	0.022659831
Proteoglycans in cancer	2.13501292	0.001049365	0.028507757
Parathyroid hormone synthesis, secretion and action	2.592227951	0.001398017	0.035057975

Supplemental Table 5: eQTL analysis

IVW					
**Gene Symbol**	**Phenotype**	**SNPs**	**Estimate (95% CI)**	**P-value**	**Heterogeneity p-value**
FST	eGFR	3	0.005 (0.002 – 0.007)	5.5E-5	0.45
SMAD7	eGFR	3	-0.002 (-0.004 – 0.0005)	0.01	0.75

A Bonferroni-corrected association p < (0.05/5 genes=0.01) was considered significant.

Supplemental Table 6: Antibodies used

Antibody Names	Company	Catalogue No
CD81 (clone:5A6)	Biolegend (San Diego, CA, USA)	349514
CD63 (clone: polyclonal)	Abcam (Cambridge, UK)	ab216130
Alix (clone: polyclonal)	Abcam	ab88388
Syntenin (clone: EPR8102)	Abcam	ab133267
58K Golgi protein (clone: 58K-9)	Abcam	ab27043
IL18 (clone: polyclonal)	Thermo Fisher Scientific (Waltham, MA, USA)	PA5-110679
LCN2 (clone ERP5084)	Abcam	ab125075
HAVCR1 (KIM1) (clone: polyclonal)	Thermo Fisher Scientific	PA5-79345
Goat anti-Rabbit IgG (clone: polyclonal)	Thermo Fisher Scientific	A-21428
Alexa Fluor 555 (clone: polyclonal)	Thermo Fisher Scientific	A-31572
Anti-Mouse IgG (clone: polyclonal)	Agilent (Santa Clara, CA, USA)	P0447
Anti-Rabbit IgG (clone: polyclonal)	Cell Signaling Technology (Danver, MA, USA)	7074S

Supplemental Table 7: Cells used for KOC and proximal tubule epithelial cell cultures

Cell Names	Company	Catalogue No	Source
hRPTEC -Primary Human Renal Proximal Tubule Epithelial Cells	Lonza (Basel, Switzerland)	CC-2553	Normal human donor tissue
hMVEC -Primary Human Glomerular Microvascular Endothelial Cells	Cell Systems (Kirkland, WA, USA)	ACBRI 128	Normal human donor tissue

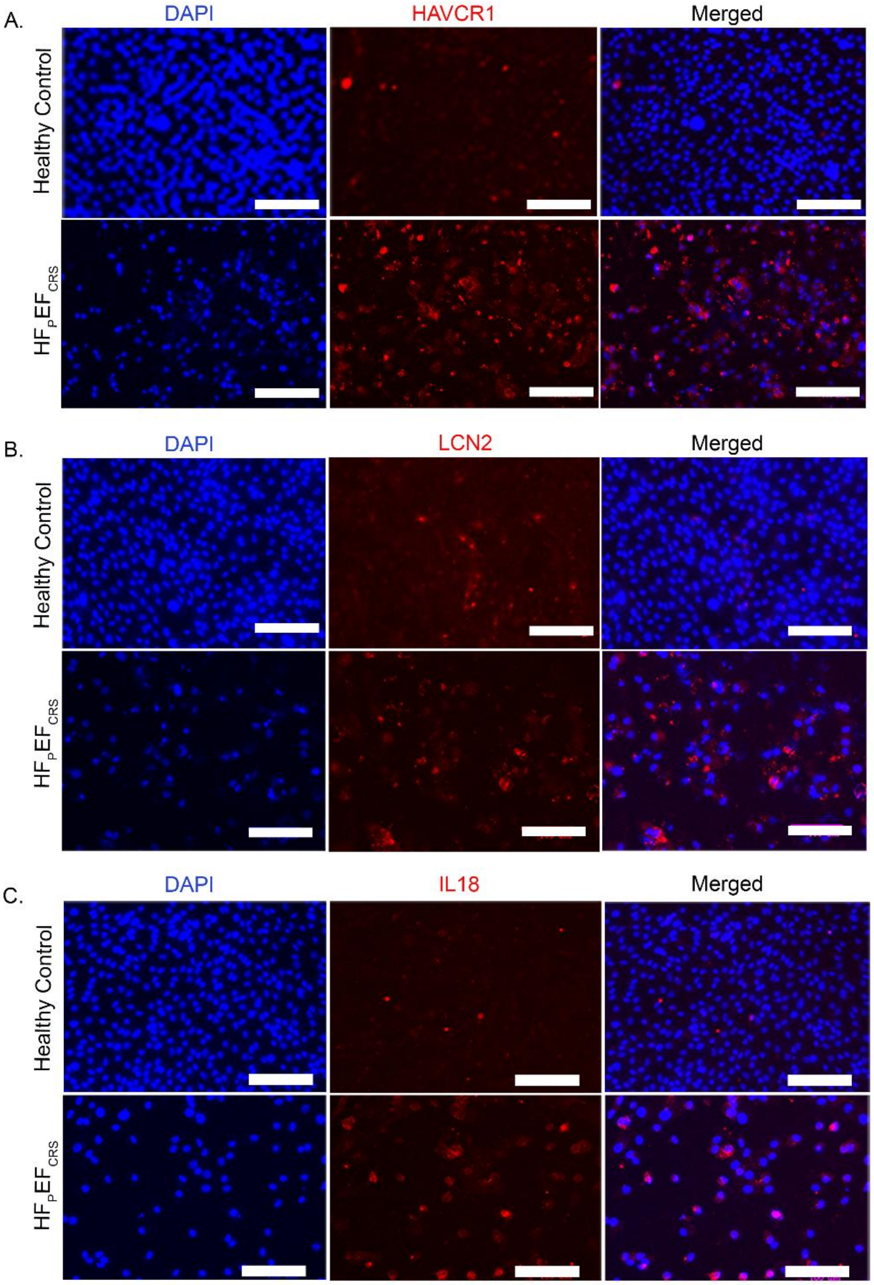

Supplemental Figure 1: Immunofluorescence study of kidney injury markers: Immunofluorescence study showing increased expression of HAVCR1, LCN2 and IL18 in the epithelial cells of HF_PEF_{CRS} groupcompared to Healthy Control group (Magnification= 100µm). Representative images of three independent experiments conducted.

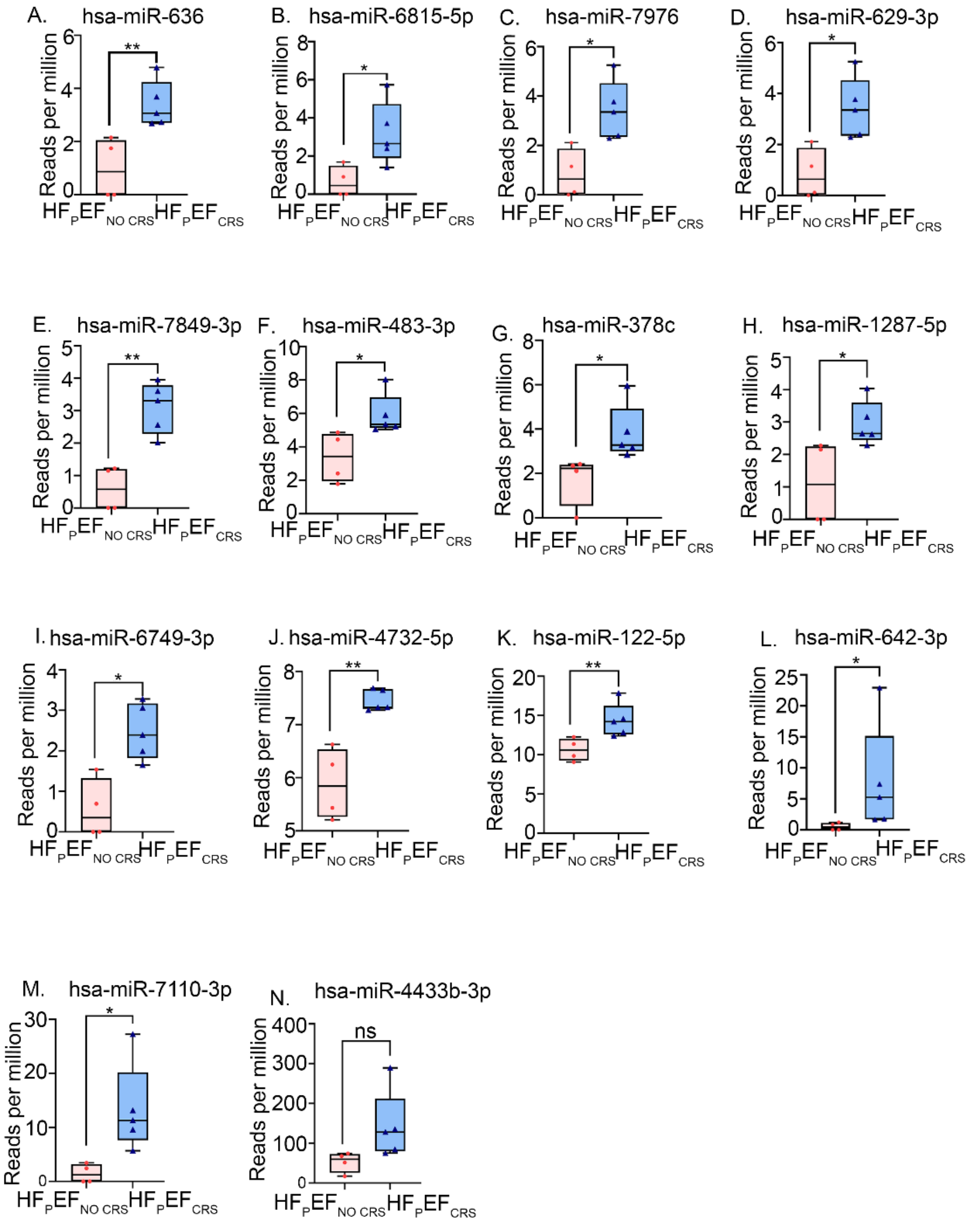

Supplemental Figure 2: (**A-N**) Box and whisker plots showing significant higher expression (reads per million) of different miRNAs in the EVs of the $\text{HFpEF}_{\text{CRS}}$ group compared to the $\text{HFpEF}_{\text{CRS}}$. Box plots represent the first quartile, median, and third quartile, with whiskers indicating minimum and maximum values. Results were analyzed with unpaired *t* test and expressed as ± SEM (n = 4 - 5) . ns, non significant; **, $p < 0.01$; ***, $p < 0.001$.

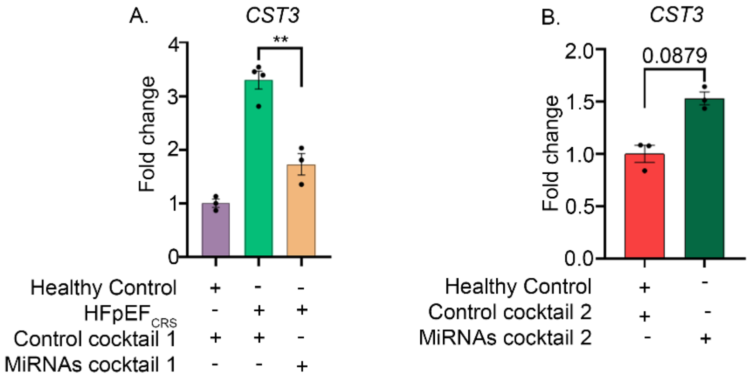

Supplemental Figure 3. MiRNA cocktail 1 and cocktail 2 attenuate or mimic the effects of HFpEF$_{CRS}$ EVs: (A) *CST3* expression was significantly decreased in the "HFpEF$_{CRS}$+miRNAs cocktail 1 treated group" compared to "HFpEF$_{CRS}$+Control cocktail 1 treated group"; n = 3-4 for each group. **(B)** *CST3* expression was markedly increased in the "Healthy Control+MiRNAs cocktail 2 treated group" compared to "Healthy Control+Control cocktail 2 treated group".; n = 3 for each group. *GAPDH* was used as internal loading control. Results were analyzed by unpaired *t* test and expressed as ± SEM of three independent experiments. **, $p < 0.01$.

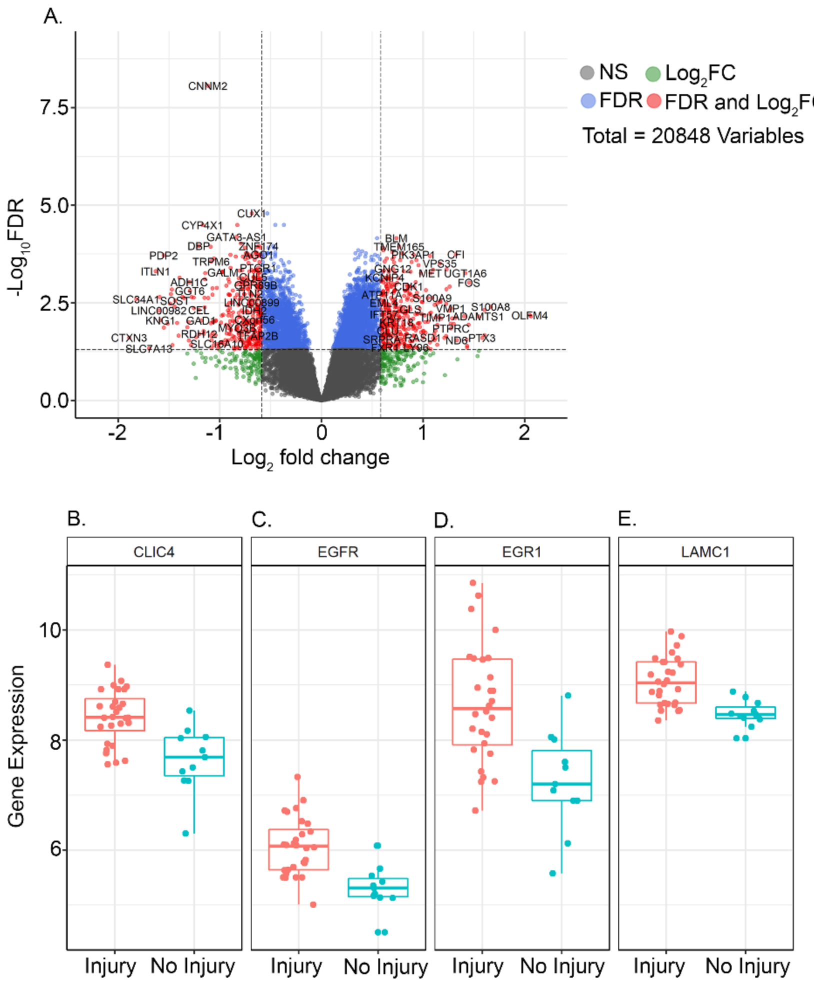

Supplemental Figure 4: (A) Volcano plot was created by all differentially expressed genes. Y axis shows Log_{10} FDR and X axis displays the log_2-fold change value. The red dots represent the differentially expressed genes with FDR adjusted p value ≤ 0.05 and absolute fold change ≥ 1.5, while green dots represent non significantly modulated genes. **(B-E)** Box and whisker plots showing differential expression of genes in the “Injury” and “No Injury” groups. Box plots represent the first quartile, median, and third quartile, with whiskers indicating minimum and maximum values.

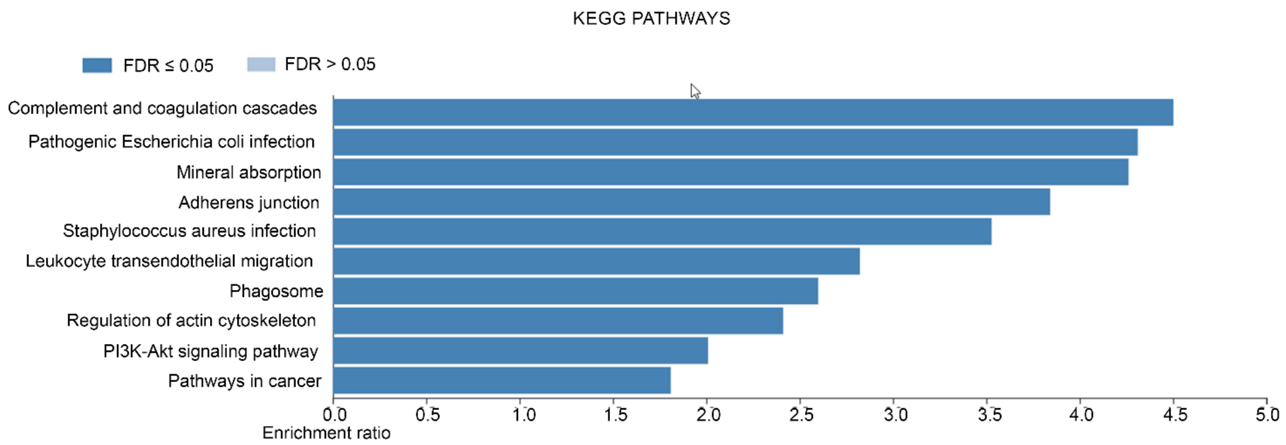

Supplemental Figure 5. Pathway analysis by WebGestalt. The enrichment bar chart is shown for the top 10 enriched KEGG pathways with FDR $\leq$ 0.05. As the enrichment increases, the corresponding function is more specific. KEGG, Kyoto Encyclopedia of Genes and Genomes; PI3K, Phosphatidylinositol 3-kinase; AKT, protein kinase.

Supplemental methods

RNAse A Treatment

Isolated EVs (210 µL) were incubated with RNAse A at a 0.5µg/µL final concentration to degrade any extracellular RNAs not protected within EVs (Thermo Fischer Scientific, Waltham, MA, USA) for 20 minutes at 37 °C followed by an addition of RNAse inhibitor (Thermo Fischer Scientific) at a 20U/µL concentration to inactivate RNAse A prior to RNA extraction.

Microfluidic resistive pulse sensing (MRPS)

EVs were diluted at 1:100 to prevent saturation of the upper limit of detection or aggregation, subjected to MRPS using the Spectradyne's nCS1 (Spectradyne,Signal Hill, CA, USA), and analyzed with both high and low sensitivity settings (NP100; voltage, 0.60 V; stretch, 46.0 mm and NP400; voltage, 0.40 V; stretch, 43.5 mm respectively). The pressure was preset at 7.0 mbar. Minimum2000 particles were analyzed for each sample.

Transmission electron microscopy (TEM) of plasma EVs

A drop containing 5 µL of purified plasma EVs (for both c-DGUC and SEC) was placed on parafilm, and a carbon-coated copper grid was placed on top of the drop for 30 minutes. Carbon-coated copper grids were previously glow discharged for 30 seconds to turn into an overall hydrophilic surface. For immune gold labeling, grids were blocked with 1% BSA in 1X PBS for 10 minutes, and IgG primary antibody anti-CD81 (Santa Cruz Biotechnology, Dallas, TX, USA; 1:30 dilution in blocking reagent) was used for 30 minutes at room temperature. Grids were then washed three times in 1X PBS and incubated with Protein A, conjugated with 10 nm gold particle (1:30 dilution) for 20 minutes at room temperature. Grids were washed twice with 1XPBS (for 5 minutes total) and 4 times with water (for 10 minutes total). Grids were stained with 0.75% uranyl formate for 1 minute and visualized on a JEOL 1400 electron microscope outfitted with an Orius SC1000 CCD camera (Gatan, Inc. Pleasanton, CA, USA).

Immunofluorescence analysis

Epithelial cells, grown on coverslips were treated with EVs from either Healthy Control or $HFpEF_{CRS}$ patients and then stained with the primary antibodies (NGAL, IL-18 and KIM-1) followed by incubation with Alexa Fluor 555-labeled secondary antibodies (Molecular Probes) (Supplemental Table 5). After the slides were mounted with Vectashield (with DAPI [4′,6-diamidino-2-phenylindole]) (Vector Laboratories, Newark, CA), the slides were examined under a fluorescence microscope (BioRad).

Long RNA sequencing of plasma EV samples

Long RNA sequencing on the exRNAs isolated from the plasma of HFpEF patients with high creatinine (n = 9, 1.1-1.6 mg/dL) and low creatinine (n=9, 0.7-0.9 mg/dL) were performed using the miRNeasy Serum/Plasma kit (Qiagen). cDNA libraries were constructed using the SMARTerStranded Total RNA-Seq Kit v2 Pico Input Mammalian (Takara Bio, San Jose, CA, USA) and sequenced using NextSeq 2000 platform.

Deconvolution analysis for the identification of source organs:

A similar approach to identify the source tissue has been previously (1) performed to generate the log transformed gene expression values for each sample in the Creatinine high and low groups, the DESeq2 (https://genomebiology.biomedcentral.com/articles/10.1186/s13059-014-0550-8) rlog function was used. Tissue-specific genes were obtained from the protein atlas website(https://pubmed.ncbi.nlm.nih.gov/25613900/), and a random sample of up to 172 tissue-specific genes (corresponding to the bottom 25% percentile of the number of tissue-specific genes across tissues) was selected for each tissue. The tissue enrichment score for each sample was calculated using the log transformed gene expression values and tissue specific genes, following the algorithm of PangoDB (https://academic.oup.com/database/article/doi/10.1093/database/baz046/5427041?login=false). This process of tissue specific gene sampling and tissue enrichment score calculation was repeated 1000 times. The median tissue enrichment score for each tissue and sample was then calculated from these 1000 repeats.

Finally, the tissues were ranked based on the median of median tissue enrichment scores of samples in the creatinine high group, and these rankings were visualized using a violin plot.

The tissue-enriched genes of adipose tissue, heart muscle, kidney, liver, pancreas and skeletal muscle were downloaded from The Human Protein Atlas (https://www.proteinatlas.org/) (2). For each tissue, the tissue-enriched genes were ranked by the RNA tissue specificity score and the top 5 genes were selected to represent the tissue. The TPM values of these genes for each sample in the Creatinine high and low groups were calculated using the convert Counts function from the DGEobj.utils package and visualized by ggplot2.

Cellular transfection of miRNAs inhibitors and mimics:

To confirm the role of miR-192-5p, 21-5p and 146a-5p in regulating the expression of kidney injury markers and TGF beta pathway related proteins, epithelial cells were treated with corresponding miRNA inhibitors and miRNA mimics in the various conditions. Epithelial cells transfected with a mixture (MiRNAs cocktail 1; Qiagen, Hilden, Germany) of synthetic miRNA inhibitors (single strand oligonucleotides) formiR-192-5p (5'-GGCTGTCAATTCATAGGTC-3'), miR-21-5p (5'-ACATCAGTCTGATAAGCT-3') and miRNA mimic for miR-146a-5p (5'-UGAGAACUGAAUUCCAUGGGUU-3') followed by treating with $HFpEF_{CRS}$ derived EVs. Both Healthy Control and another group of $HFpEF_{CRS}$ derived EVs treated cells were pretreated with a mix (Control cocktail 1) of LNA miRNA Inhibitor Controls (Qiagen) and scrambled NC siRNA (All Stars Negative Control siRNA, Qiagen) (Figure 10A).

On the other hand, one group of epithelial cells pretreated with a mixture (MiRNAs cocktail 2) of synthetic miRNA mimics for miR-192-5p (5'-CUGACCUACGAAUUGACAGCC-3'), miR-21-5p (5'-UAGCUUAUCAGACUGAUGUUGA-3') and inhibitor for miR-146-5p (5'-ACCCATGGAATTCAGTTCTC-3') followed by treatment with Healthy Control derived EVs and other group, pretreated with Control cocktail 2 (LNA miRNA Inhibitor Control and scrambled NC siRNAs, Qiagen) further treated with Healthy Control derived EVs (Figure 11A). Cells were maintained for 72 hours. All transfections were performed using Lipofectamin RNAiMax transfection Reagent (Invitrogen, Waltham, MA, USA) following manufacturer instructions.

References cited for supplemental methods:

1. Gokulnath P, Spanos M, Lehmann HI, Sheng Q, Rodosthenous R, Chaffin M, et al. Plasma extracellular vesicle transcriptome as a dynamic liquid biopsy in acute heart failure. *medRxiv.* 2023.

2. Uhlén M, Fagerberg L, Hallström BM, Lindskog C, Oksvold P, Mardinoglu A, et al. Proteomics. Tissue-based map of the human proteome. *Science.* 2015;347(6220):1260419.

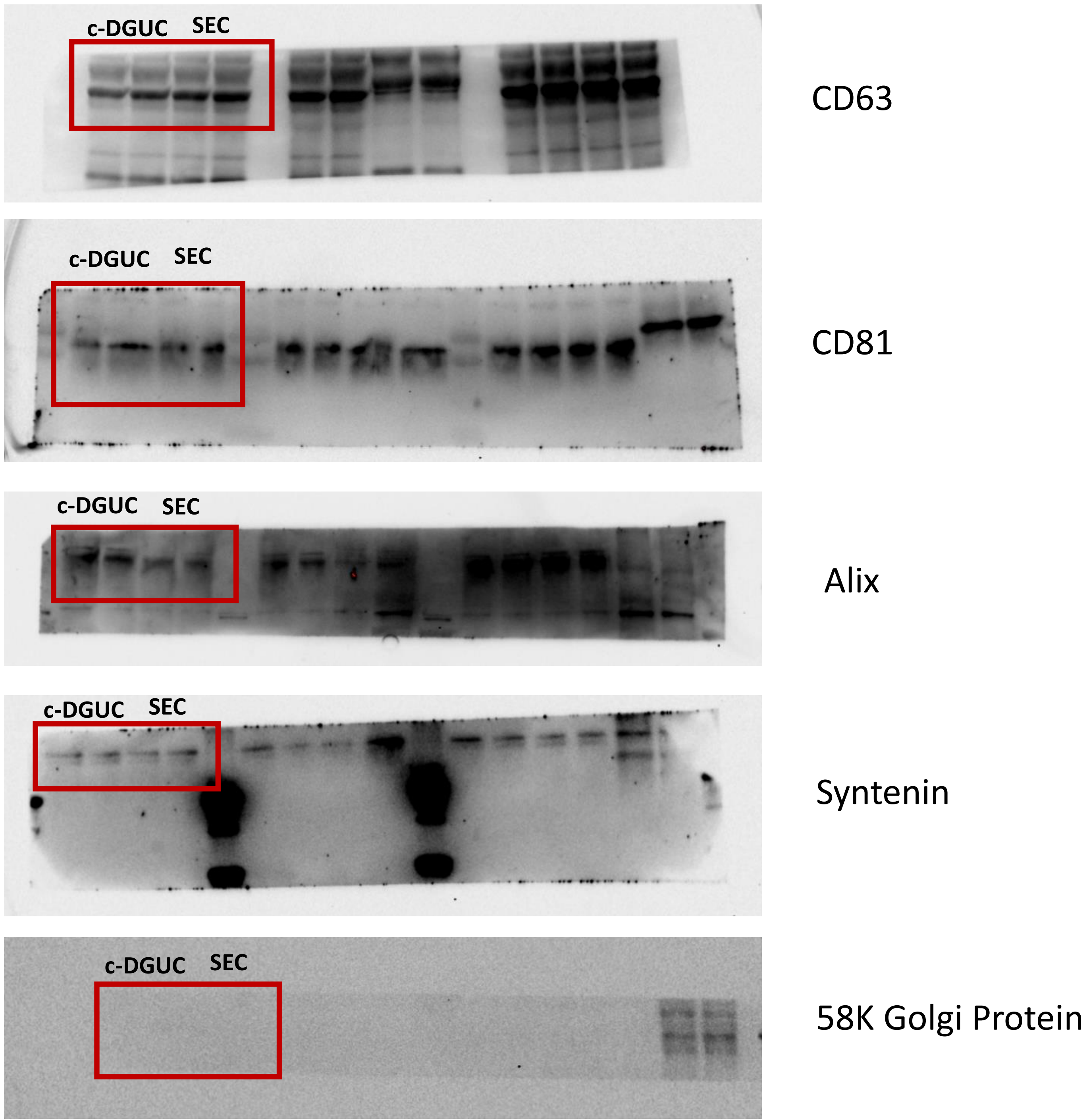
c-DGUC
SEC
CD63
c-DGUC
SEC
CD81
c-DGUC
SEC
Alix
c-DGUC
SEC
Syntenin
c-DGUC
SEC
58K Golgi Protein
